# Solution-Processed Multiferroic Thin-Films with Large Magnetoelectric Coupling at Room-Temperature

**DOI:** 10.1021/acsnano.2c09769

**Published:** 2023-04-17

**Authors:** Hamed Sharifi Dehsari, Morteza Hassanpour Amiri, Kamal Asadi

**Affiliations:** † Max Planck Institute for Polymer Research, Ackermannweg 10, 55128 Mainz, Germany; ‡ Centre for Therapeutic Innovations, University of Bath, Claverton Down, BA2 7AY Bath, United Kingdom; § Department of Physics, University of Bath, Claverton Down, BA2 7AY Bath, United Kingdom

**Keywords:** multiferroic, ferroelectric polymer, magnetic nanoparticle, nanocomposites, magnetoelectric coupling

## Abstract

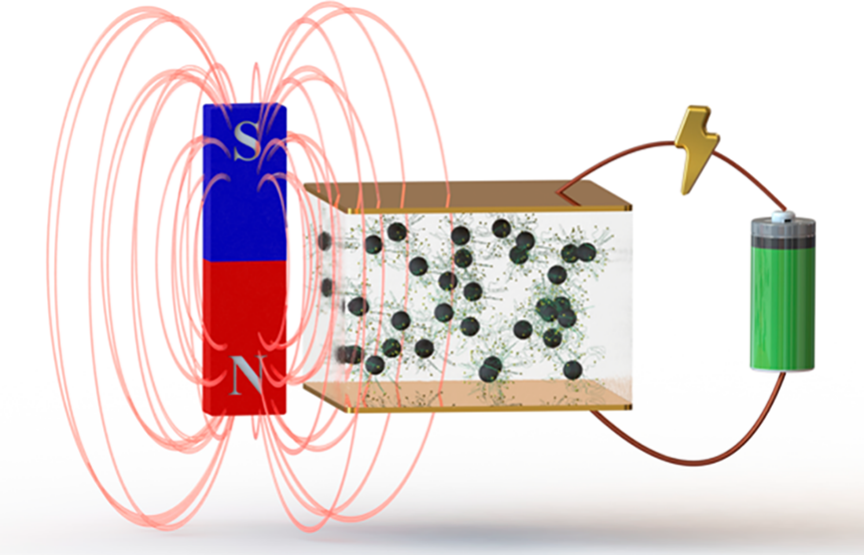

Experimental realization of thin films with a significant room-temperature magnetoelectric coupling coefficient, *α*_ME_, in the absence of an external DC magnetic field, has been thus far elusive. Here, a large coupling coefficient of 750 ± 30 mV Oe^–1^ cm^–1^ is reported for multiferroic polymer nanocomposites (MPCs) thin-films in the absence of an external DC magnetic field. The MPCs are based on PMMA-grafted cobalt-ferrite nanoparticles uniformly dispersed in the piezoelectric polymer poly(vinylidene fluoride-*co*-trifluoroethylene, P(VDF-TrFE). It is shown that nanoparticle agglomeration plays a detrimental role and significantly reduces *α*_ME_. Surface functionalization of the nanoparticles by grafting a layer of poly(methyl methacrylate) (PMMA) *via* atom transfer radical polymerization (ATRP) renders the nanoparticle miscible with P(VDF-TRFE) matrix, thus enabling their uniform dispersion in the matrix even in submicrometer thin films. Uniform dispersion yields maximized interfacial interactions between the ferromagnetic nanoparticles and the piezoelectric polymer matrix leading to the experimental demonstration of large *α*_ME_ values in solution-processed thin films, which can be exploited in flexible and printable multiferroic electronic devices for sensing and memory applications.

## Introduction

The magnetoelectric coupling coefficient, *α*_ME_, characterizes the strength of magnetoelectric interaction  (in mV Oe^–1^cm^–1^), where *V* is the voltage induced by a time-varying (AC) magnetic field, *H*_AC_, across the multiferroic film with thickness, *t*. Single-phase multiferroic materials are rare and exhibit small *α*_ME_ values of just a few mV Oe^–1^ cm^–1^, usually observed at low temperatures.^1−3^ Biphase multilayers have been suggested to increase the coupling coefficient.^4−9^ The magnetoelectric coupling arises from interfacial stress–strain transfer between the ferroic phases.^4^ Thick films of piezoelectric polymers deposited on highly magnetostrictive materials, hence bilayer heterostructures, show relatively large *α*_ME_ values of several thousands of mV Oe^–1^ cm^–1^. For many of the envisioned applications of the multiferroics composites in sensors, memories, tunable filters, and signal processing technologies, thin films are needed, which enable miniaturization and integration of the multiferroic devices with the semiconductor optoelectronic devices. However, thin films of bilayer heterostructures exhibit weak magnetoelectric coupling and small *α*_ME_ values due to substrate clamping and thus not suited for the envisioned applications.^9^

Polymer-based multiferroic composites (MPCs) comprising a piezoelectric polymer with magnetic nanoparticle fillers have been theoretically proposed as a promising route toward obtaining significant coupling coefficients comparable to the bilayer heterostructures.^10^ Values for *α*_ME_ as high as a thousand mV Oe^–1^cm^–1^ have been predicted for MPCs at room temperature,^11^ which, if realized in thin films, would render the MPCs viable for integrating with microelectronic devices. The flurry of activities on realizing MPCs has been typically centered around mixing a piezoelectric polymer such as poly(vinylidene fluoride), PVDF, or its random copolymer with trifluoroethylene, P(VDF-TrFE), with various magnetic nanoparticles.^12^ Besides the theoretical prediction of large coupling coefficients, MPCs can enable low-temperature solution processing of flexible devices,^39,40^ which starkly contrasts with brittle ceramic-based multiferroics.^41^ In sharp contrast with the theoretical predictions, coupling coefficients of just a few mV Oe^–1^ cm^–1^ have been experimentally reported, as summarized in Table S1. As it stands, the simple approach of mixing the piezoelectric polymer with different magnetic nanoparticles, regardless of their type and magnetic properties, has so far produced only weak *α*_ME_ values that are measured only when an external DC magnetic field is applied and only when a thick-films of several tens of micrometers is used. Large *α*_ME_ values in thin films of MPCs in the absence of an external DC magnetic field, *i*.*e*., a self-biased MPC, are yet to be reported.

We suggest that the lack of MPC thin films and the reported low coupling coefficient for MPCs thick films are due to the agglomerations of magnetic nanoparticles in the piezoelectric polymer matrix. The reported theoretical predictions outline, although implicitly, that the prerequisite for achieving a significant *α*_ME_ coupling coefficients value in the MPCs is a uniform dispersion of isolated magnetic nanoparticles.^11^ A careful inspection of the structural analysis for the reported MPCs (Table S1) reveals that a uniform distribution of the individual nanoparticles in the piezoelectric matrix is lacking in all reports.

MPCs are typically fabricated by mixing some types of magnetic nanoparticles with the PVDF or P(VDF-TrFE) in the solution phase. The nanoparticles tend to agglomerate due to their high surface area and van der Waals interactions.^13^ For nonmagnetic nanoparticles, interparticle interactions are mitigated using well-established and relatively simple interfacial modifications by small molecular organic surfactants (or ligands) such as oleic acid. For the magnetic nanoparticles, the agglomeration tendency is enhanced *via* the magnetic interactions, which are operational on larger length scales than the length of the ligand. For the reported MPCs (see, for instance, the references in Table S1), conventional surface modification using a small organic ligand only renders the nanoparticle colloidally stable in the solution phase, whereas obtaining a uniform dispersion in the matrix requires compatibilization of the nanoparticles with the piezoelectric polymer matrix. Since the bare nanoparticles or those modified with small organic ligands are immiscible with the polymer matrix, upon processing the composite either from solution phase or melt, phase separation sets in, and large clusters of agglomerated nanoparticles are inevitably formed. The aggregation is facilitated further by the interparticle magnetic interactions. Besides reducing *α*_ME_ value, the agglomeration also increases the dielectric loss and the leakage current through the composite, thereby impeding a reliable evaluation of *α*_ME_.^14^ Circumventing agglomeration and achieving uniform dispersion of the magnetic nanoparticle is, therefore, the key to obtaining large *α*_ME_ in MPC thin film and should be addressed.

Here we first elucidate by finite element analysis the origin of magnetoelectric coupling in MPCs and substantiate the detrimental effect of agglomeration on the coupling coefficient. We put forth material design considerations to achieve a self-biased MPC with large *α*_ME_ values. Uniform dispersion of the nanoparticles, Co_0.7_Fe_2.3_O_4_, in P(VDF-TrFE) matrix in thin films is achieved by avoiding agglomeration through grafting poly(methyl methacrylate) (PMMA) chains from the surface of the nanoparticles. The choice of PMMA as a nanoparticle surfactant is rationalized and substantiated by its complete miscibility over the entire composition range with P(VDF-TrFE), which renders the nanoparticle miscible in a P(VDF-TrFE) matrix and avoids phase separation. Through optimizing composition, we demonstrate a self-biased MPC with large *α*_ME_ values of 750 ± 30 mV Oe^–1^ cm^–1^, which is significantly larger than the reported values for MPC thin films.

## Results and Discussions

### Operation Mechanism and the Detrimental Effect of Agglomeration

**Figure 1:**
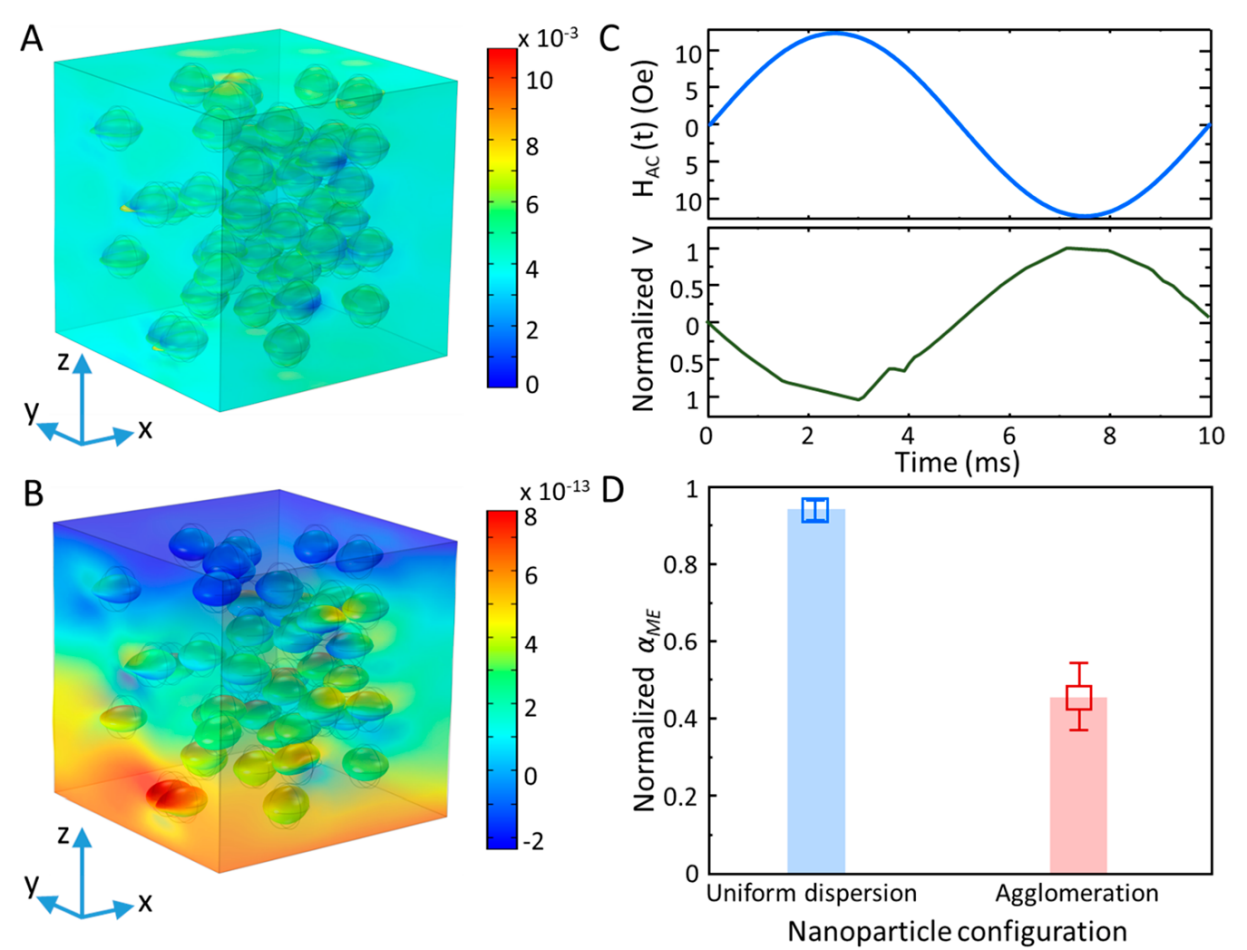
Origin of the reduced coupling coefficient in MPCs. (A and B) Simulation of the MPC with uniform nanoparticle dispersion under an AC magnetic field, *H*_AC_, that varies along the *z*-axis. For clarity, the strain in the nanoparticles has been graphically magnified to show the effect of magnetostriction. The volumetric stress in the piezoelectric phase by the strained nanoparticles is shown in (A), and the resulting voltage in the piezoelectric phase is shown in (B). (C) Calculated magnetically induced voltage is out of phase with the *H*_AC_ because of the negative *d*_33_ coefficient of P(VDF-TrFE). (D) Comparison between the voltages generated by MPCs with fine uniform dispersion and agglomerated nanoparticles. For every scenario, the statical average is obtained for at least 10 simulations with different nanoparticle distributions.

To substantiate the operational mechanism of the MPCs and the adverse effect of agglomeration, finite-element method simulations have been performed for both scenarios of uniform and agglomerated dispersions of magnetic nanoparticles (cobalt-ferrite in this case) in a piezoelectric polymer matrix made of P(VDF-TrFE). The ME coupling is closely correlated with the effective magnetostriction coefficient of the nanoparticles, piezoelectric strain coefficient, elastic moduli of the matrix, and strain transfer efficiency. Cobalt-ferrite, with Co stoichiometry of 0.7, hence Co_0.7_Fe_2.3_O_4_, is chosen for its relatively large magnetostriction coefficient, which approaches 590 × 10^–6^ along the [100] direction.^15^ The choice of P(VDF-TrFE) as the matrix is motivated by its sizable piezoelectric voltage coefficient of −0.372 V m N^–1^. The code is generic and can be adapted to different materials systems. Details of the simulation are given in the Supporting Information. An MPC thin film, schematically shown in Figure 1A, is subjected to a time-varying magnetic field, *H*_AC_, along the *z*-axis. The induced strain in the nanoparticles as a function of *H*_AC_, the volumetric stress exerted on the piezoelectric phase, and the voltage difference developed across the capacitor plates are calculated. The graphics in Figure 1A,B present a snapshot of the calculations when *H*_AC_ is maximum for uniform dispersion of the nanoparticles. As *H*_AC_ varies, the nanoparticles strain and stress the nearby piezoelectric phase. The strained nanoparticles (Figure 1A) stress the nearby piezoelectric phase. The resulting volumetric stress in the P(VDF-TrFE) phase generates a piezo-voltage (Figure 1B) that varies in time and is out-of-phase with the applied *H*_AC_ (Figure 1C) because P(VDF-TrFE) has a negative piezoelectric voltage coefficient.

Following establishing the operational mechanism, *α*_ME_ for the composites with uniform and agglomerated nanoparticle dispersion are calculated. Agglomeration of the nanoparticles severely reduces the interfacial area, as shown in Figure S1, thereby reducing strain transfer between the two phases. Typical examples of the structures used for the simulations of the MPCs with uniform dispersion and aggregated nanoparticles are given in Figure S2. Details of the models are given in the Supporting Information and Figures S2–S4. The summary of the calculated coupling coefficients, presented in Figure 1D, unambiguously shows that the MPCs with uniform nanoparticle dispersion have a larger *α*_ME_ with a narrow standard deviation from its mean value. In contrast, the composite with agglomerated particles has a significantly smaller *α*_ME_ with a broader standard deviation from the mean value. The reduction in *α*_ME_ is solely due to the agglomeration because the stress is transferred to the nearby nanoparticles instead of the piezoelectric phase. Hence, magnetostrictive strain is effectively damped. The interfacial interaction between the magnetic and piezoelectric phases is reduced, yielding a reduced piezo voltage and, therefore, substantially reduced *α*_ME_ values.

The simulations suggest that, for the experimental realization of the MPC thin films with large *α*_ME_ in the absence of an external DC magnetic field, *i*.*e*., a self-biased multiferroic composite, first, highly magnetostrictive nanoparticles and polymers with large piezoelectric voltage coefficients should be used. Second, the interfacial area between the magnetic nanoparticle and the piezoelectric polymer phases should be maximized, which means that the size of the nanoparticles should be reduced to the nanosize regime. Third, the particles should possess room-temperature remanent magnetization to enable the realization of a self-biased multiferroic. Lastly, but most importantly, the nanoparticle should be compatibilized with the polymer matrix to ensure uniform dispersion, thus maximizing the interfacial magnetoelectric coupling interactions.

### Downsizing and Compatibilization of Cobalt-Ferrite Nanoparticles

Thermal decomposition is used to synthesize cobalt-ferrite, Co_*x*_Fe_3–*x*_O_4_, nanoparticles, and the size and stoichiometry of the nanoparticles are finely tuned through fine-tuning of the synthesis conditions. The Co_*x*_Fe_3–*x*_O_4_ nanoparticles are chosen due to their relatively large magnetostriction coefficient^15^ and large magnetocrystalline anisotropy,^15,16^ which enables nanosizing the particles while maintaining the room-temperature remanent magnetization.^17,18^ However, a complex interplay exists between Co stoichiometry, *x*, magnetic properties, and particle size, which should be investigated to find the optimized stoichiometry range.

**Figure 2:**
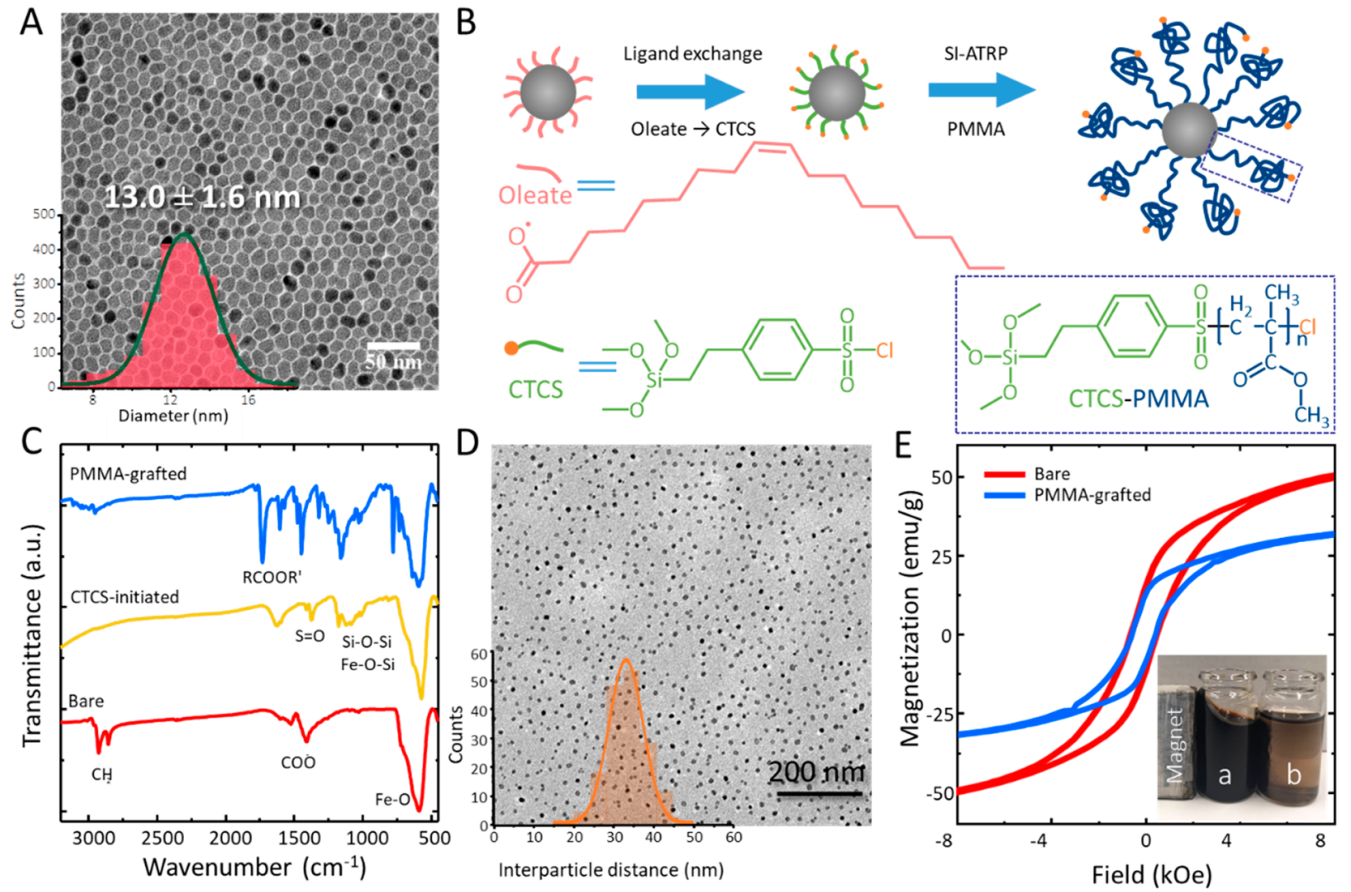
Compatibilization of cobalt-ferrite nanoparticles. (A) TEM image of bare (oleate-coated) cobalt-ferrite nanoparticles. The inset is the size distribution histogram obtained for more than 2000 nanoparticles. (B) Schematic representation of SI-ATRP synthesis of PMMA on the nanoparticles. (C) FTIR spectra of oleate-, CTCS-, and PMMA-coated cobalt-ferrite nanoparticles. (D) TEM image of the PMMA-coated cobalt ferrite nanoparticle with PMMA molecular weight of *M*_n_ = 41 kg/mol. The inset shows the histogram of the center-to-center distance between PMMA-grafted nanoparticles. (E) Room temperature magnetization as a function of applied magnetic field for bare and PMMA-grafted cobalt ferrite nanoparticles with an average diameter of 13 ± 1.6 nm and Co stoichiometry, *x* ∼ 0.7. The inset shows the colloidal stability of (a) PMMA-grafted and (b) as-synthesized oleate-coated nanoparticles in an organic solution (toluene) in the presence of the magnetic field.

A typical TEM image of the as-synthesized nanoparticles and their size distribution are given in Figure 2A. Through an extensive synthesis attempt combined with the characterization of the magnetic properties of the nanoparticles, details we have published previously,^19^ it is found that the optimum cobalt stoichiometry, *x*, is 0.7, which enables reducing the size for the nanoparticle with stable room-temperature magnetization to just 13 ± 1.6 nm (in diameter).^19^ The nanoparticles presented in Figure 2A possess a stable room-remanent magnetization, *M*_r_, that amounts to 25 emu g^–1^, as shown in Figure 2E.

The next step is the compatibilization of the Co_0.7_Fe_2.3_O_4_ nanoparticles with the piezoelectric polymer matrix, P(VDF-TrFE). Compatibilization is realized by grafting poly(methyl methacrylate) (PMMA) on the surface of the nanomagnets. Surface-initiated atom transfer radical polymerization (SI-ATRP) is chosen to grow the PMMA chains from the nanoparticles’ surface, as shown in Figure 2B, because SI-ATRP enables precise control over the thickness of the PMMA shell.^20−23^

Fourier transform infrared spectroscopy (FTIR) (Figure 2C) shows the presence of CH and COO bands, indicating that the as-synthesized nanoparticles are capped with oleate surfactant. Therefore, the first step in our SI-ATRP is to replace the oleate with the surface initiator 2-(4-chlorosulfonylphenyl) ethyltrichlorosilane (CTCS). Details of the ligand exchange procedure are described in the Methods section. The FTIR spectra of the nanoparticles after the ligand exchange process (Figure 2C) demonstrate the successful replacement of CTCS. Subsequently, PMMA growth is initiated *via* ultrasound-mediated ATRP. Details of the polymerization are given in the Supporting Information. The FTIR spectra of PMMA-grafted nanoparticles (Figure 2C) show the characteristic peak of the ester group of PMMA at 1730 cm^–1^, confirming the successful growth of PMMA on the surface.^22,23^ The kinetics of SI-ATRP on cobalt-ferrite nanoparticles, given in Figure S5, confirms that the polymerization proceeds in a living fashion. The success of ATRP in grafting a PMMA shell on the surface of the nanoparticles is demonstrated in Figure S6. ATRP provides a high level of control over the kinetics of the reaction; hence, the thickness or molecular weight of the PPMA shell could be precisely controlled with a polydispersity (PDI) that varies between 1.1 and 1.25.

The PMMA chains with low molecular weight may not be able to efficiently avoid both types of agglomeration, as shown in Figure S8. On the other hand, PMMA chains with high molecular weight negatively affect the performance of the composite because, in a fixed weight of the composite, the addition of such PMMA-grafted magnetic nanoparticles increases the volume fraction of the nonfunctional PMMA. Hence, we chose an intermediate molecular weight of 41 kg mol^–1^ for the rest of the study. It should be noted that, for the chosen molecular weight, the PMMA chains are in the concentrated polymer brush regime (CPB), where all the chains are stretched (see Figure S9 and the discussion in the Supporting Information). A TEM image of the grafted nanoparticles with a PMMA molecular weight of *M*_n_ = 41 kg mol^–1^ is shown in Figure 1D. Magnetically induced agglomeration is entirely prevented. The histogram of center-to-center distances for the PMMA-grafted nanoparticles in Figure 1D shows that the nanoparticles are separated by the PMMA shell, with a 34 ± 5.5 nm distance. Experimentally, the *M–H* curve of the PMMA-grafted nanoparticles (Figure 2E) shows a reduction of *M*_s_ (obtained at 20 kOe) from 64.5 emu gr^–1^ for the bare to 42.3 emu gr^–1^ of the PMMA-grafted nanoparticles. The reduction of remanent magnetization is due to the presence of the PMMA shell, which constitutes nearly 37% of the total weight of the grafted nanoparticles (determined from TGA, Figure S7). Therefore, the *M*_s_ of the bare nanoparticles should be reduced by the same factor to 40.6 emu gr^–1^, which is very close to the experimentally measured value. The growth of the PMMA shell very efficiently hinders particle agglomeration in solution and leads to a high degree of colloidal stability of the nanoparticles in organic solvents such as toluene and cyclopentanone even in the presence of a strong magnetic field, as presented in the inset of Figure 2E.

### Thin-Film Fabrication and Characterization

**Figure 3:**
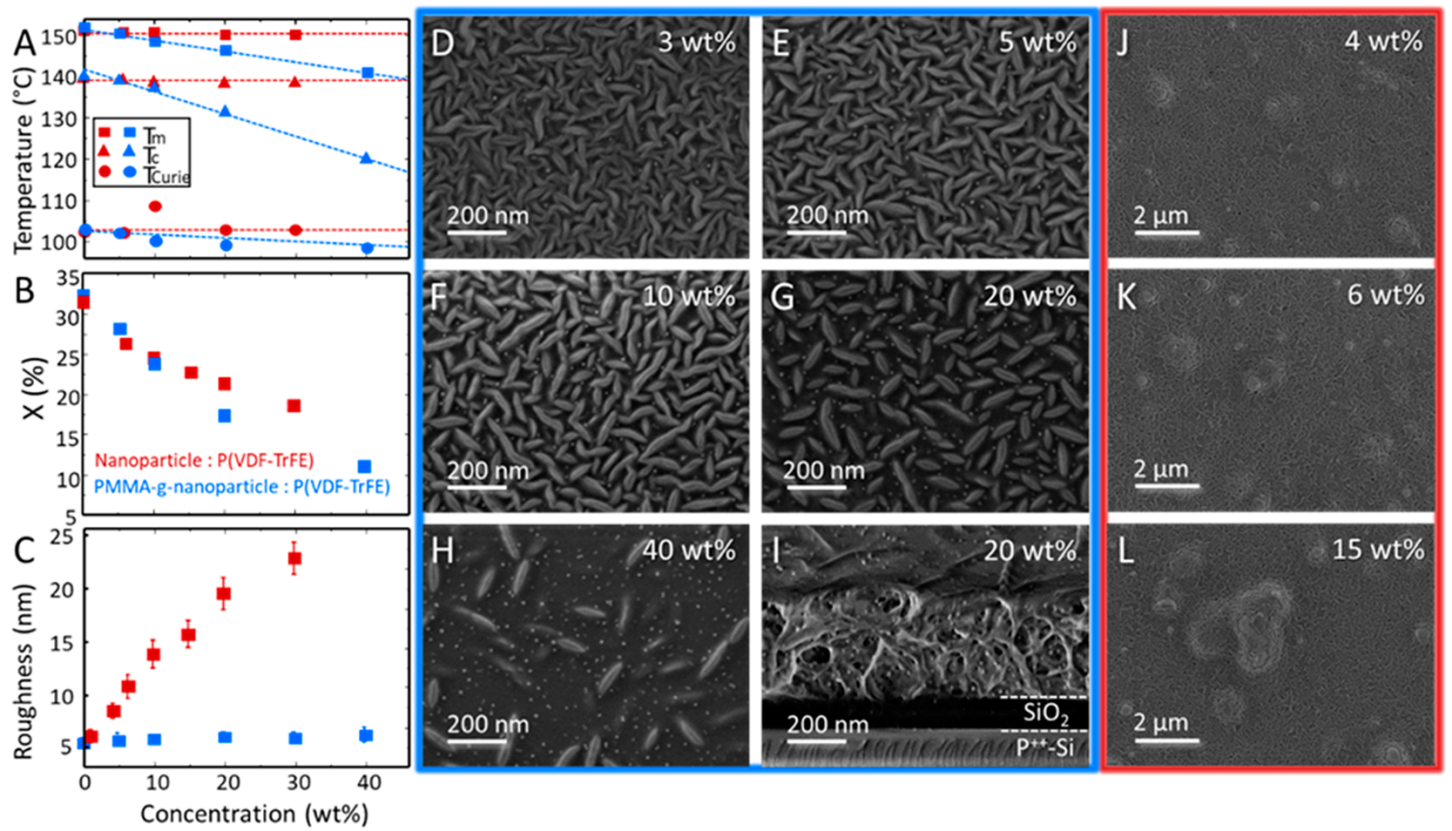
Microstructure of the polymer multiferroic thin films. (A) Evolution of the melting (*T*_m_), crystallization (*T*_c_), and Curie (*T*_Curie_) temperatures of the nanocomposite. (B) Normalized crystallinity of P(VDF-TrFE) phase as a function of nanoparticle concentration for as-synthesized (red) and PMMA-grafted (blue) nanoparticles. The dashed lines in panel A are guides to the eye. (C) Changes in the roughness of the nanocomposite thin films as a function of particle loading. (D–H) SEM topographical images for the nanocomposites of P(VDF-TrFE) with various loading of PMMA-grafted nanoparticles. (I) Cross-sectional image of a 400 nm thick P(VDF-TrFE) nanocomposite thin-film with 20 wt % loading of PMMA-grafted nanoparticle. (J–L) Nanocomposites of P(VDF-TrFE) with as-synthesized nanoparticles.

The PMMA-grafted and the synthesized oleate-coated as-synthesized nanoparticles are dispersed in P(VDF-TrFE) in the solution phase, and thin-film MPCs are fabricated from both solutions. Thin films with as-synthesized nanoparticles are fabricated as the benchmark composite. Differential scanning calorimetry (DSC) is performed to evaluate the miscibility of the nanoparticles with P(VDF-TrFE) for both composite films, and the first heating and cooling thermograms (Figure S10) are analyzed. We noted that cobalt ferrite magnetic nanoparticles do not show any phase change or a magnetic phase transition for the investigated temperature range.^24^ Therefore, the DSC curves only account for the phase transition in P(VDF-TrFE). For the benchmark composites with the as-synthesized oleate-coated nanoparticles (Figure 3A), the melting (*T*_m_) and crystallization (*T*_c_) temperatures do not show significant variation with the composition and remain constant, which indicates the immiscibility of the oleate-coated nanoparticles with the P(VDF-TrFE) matrix. In sharp contrast, the nanocomposites with PMMA-grafted nanoparticles show a monotonic depression in both *T*_m_ and *T*_c_ of P(VDF-TrFE) with increasing particle loading, indicating miscibility of the PMMA-grafted nanoparticles with the P(VDF-TrFE) matrix over the investigated composition range.^25^ The miscibility arises from the dipole/dipole interaction between the —CF_2_ groups of VDF and the —C=O groups of PMMA and the hydrogen bonding between the double-bonded oxygen of the carbonyl and the acidic hydrogen of the —CH_2_—CF_2_— group.^25^ The normalized crystallinities of various nanocomposites are presented in Figure 3B. The crystallinities are obtained from DSC thermograms and are corrected for the weight fraction of the P(VDF-TrFE) phase and then normalized to the crystallinity of the pristine P(VDF-TrFE) film, which is 35%. Due to their miscibility, the crystallinity of the nanocomposite with PMMA-grafted nanoparticles reduces much faster than that of the benchmark composite as the concentration increases.

The DSC results show that phase separation and, consequently, agglomeration are expected for the benchmark nanocomposite, whereas a uniform dispersion of PMMA-grafted nanoparticles in P(VDF-TrFE) is anticipated. The scanning electron microscope (SEM) image for thin films of pristine P(VDF-TrFE) is presented in Figure S11. Those of the nanocomposite with various loading of PMMA-grafted nanoparticles are presented in Figure 3D–H. P(VDF-TrFE) has a distinct and well-known semicrystalline microstructure. The needle-like grains of P(VDF-TrFE) crystallites are randomly distributed in the film. Hence, the needle-like grains observed in the composite thin films are the P(VDF-TrFE) crystallites. The white dots appearing in the images for the nanocomposite are the cobalt ferrite cores of the PMMA-grafted nanoparticles. The appearance of white spots is because of the higher average atomic number of the cobalt ferrite core compared with its surrounding polymer phase. Moreover, numerical image analysis reveals that the size of the white spots is nearly 11.8 ± 3.1 nm, which is similar to that of the cobalt ferrite core. The difference in the average size obtained from TEM and SEM image analysis is due to the lower contrast of the SEM micrographs. Achieving high contrast in polymeric samples is generally challenging because of the charging effects.

SEM micrographs in Figure 3D–H reveal that the P(VDF-TrFE) crystallites are pure, and the PMMA-grafted nanoparticles are accommodated in the amorphous phase of the matrix at the expense of a reduction in the size and number of the P(VDF-TrFE) crystallites, which is consistent with the crystallinities that are obtained from DSC thermograms. The SEM analysis undoubtedly confirms that the PMMA-grafted nanoparticles are uniformly distributed in the amorphous phase of the P(VDF-TrFE) matrix. The uniform dispersion is obtained for all compositions, even for loading so high as 40 wt %, as shown in Figure 3G. A cross-sectional SEM image of the sample with 20 wt % PMMA-grafted nanoparticles (Figure 3I) shows the absence of nanoparticle agglomeration in the volume of the nanocomposite thin-film that is just 400 nm thick and confirms the effectiveness of PMMA shell in preventing phase separation.

In sharp contrast, the composites with as-synthesized nanoparticles show severe particle agglomeration (Figure 3J–L) even at loading as low as 4 wt %. All benchmark composite films suffer from agglomeration, which hampers the fabrication of smooth thin films needed to evaluate the magnetoelectric coupling. The composite thin films fabricated from as-synthesized nanoparticles are rough, as shown in Figure 1C, because of the agglomeration despite using the optimal thin-film processing conditions.^32^ The attribution of roughness to agglomeration is justified by observing that agglomeration and roughness increase with particle loading. In contrast, the thin films fabricated with PMMA-grafted nanoparticles under the same processing conditions show an RMS roughness well below 10 nm and are very smooth due to the absence of agglomeration.

### Evaluation of Multiferroic Properties

**Figure 4:**
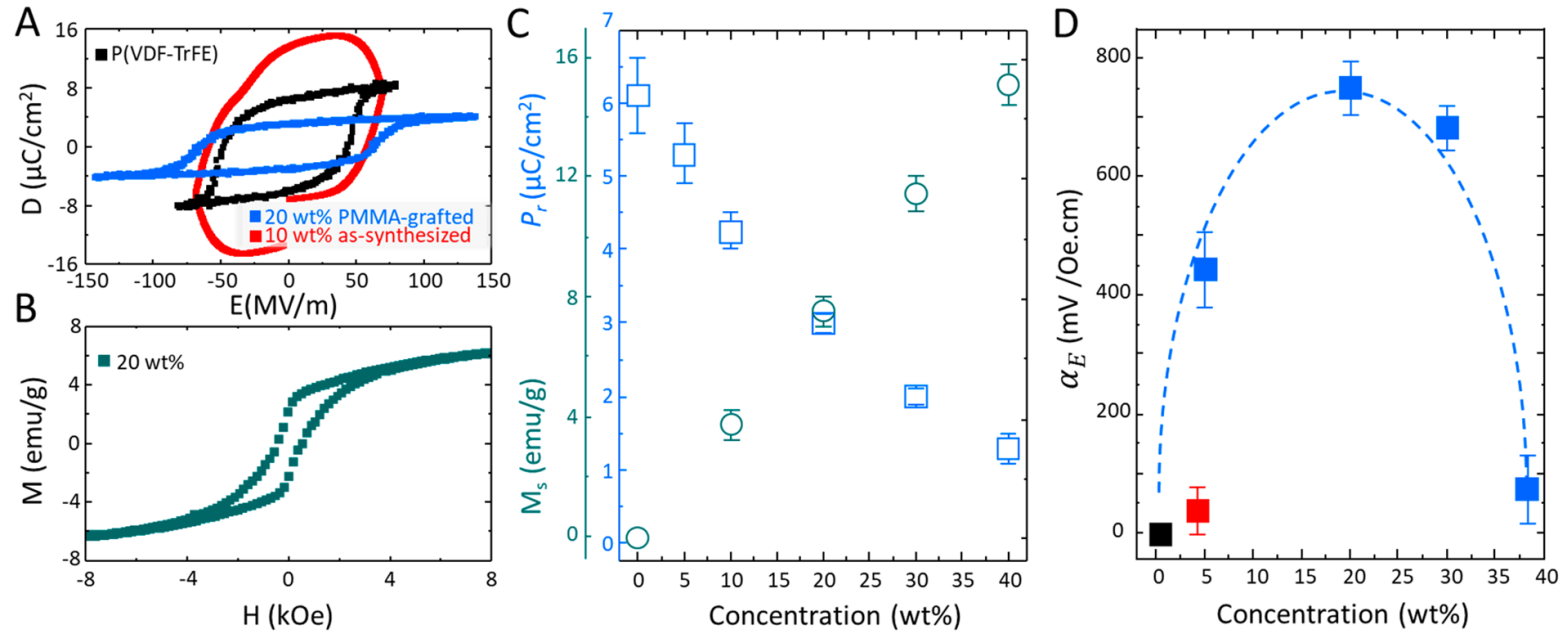
Solution-processed nanocomposite multiferroic thin films. (A) Representative ferroelectric displacement loop for a pristine P(VDF-TrFE) capacitor (black) and for P(VDF-TrFE) nanocomposites with 10 wt % as-synthesized (red) and 20 wt % PMMA-grafted nanoparticles (blue). (B) Magnetization curve for the nanocomposite of P(VDF-TrFE) with 20 wt % PMMA-grafted nanoparticles. (C) Evolution of remanent polarization, *P*_r_, and saturation magnetization, *M*_s_, of the composites as a function of the weight of PMMA-grafted nanoparticle. (D) Room-temperature magnetoelectric voltage coefficient of the nanocomposites with various loading of PMMA-grafted (blue) and as-synthesized cobalt-ferrite nanoparticles (red). The coupling coefficients are obtained under similar *H*_AC_ and frequency of 1 Oe and 10 kHz, respectively. The dashed blue line is given as a guide.

Having solved the agglomeration issue and achieved smooth thin films with a uniform dispersion of the nanoparticles in the P(VDF-TrFE) matrix, we have fabricated MPC thin-film capacitors to evaluate the ferroelectric and ferromagnetic properties. First, pristine P(VDF-TrFE) thin-film capacitors are evaluated. The coercive field, *E*_C_, and remanent polarization, *P*_r_, of the pristine P(VDF-TrFE) film (Figure 4A) amounts to 50 MV m^–1^ and 6.1 μC m^–2^, respectively. Subsequently, the benchmark nanocomposite thin-film capacitors with as-synthesized nanoparticles are measured. A representative *D*–*E* loop for the composite with just 10 wt % loading of as-synthesized nanoparticles is presented in Figure 4A. Despite being less crystalline than the pristine P(VDF-TrFE), the composite with as-synthesized nanoparticles seemingly shows increased *P*_r_ and *E*_C_, which is not physical and is due to the increased leakage current through the device, as explained by Scott in his famous paper about ferroelectricity in bananas.^26^ Please note that ferroelectric displacement loops implicitly contain information about AC leakage current, which can be obtained by differentiating the measured displacement with respect to time. Due to the increased AC conductivity and losses, larger applied fields are required to switch the polarization of a leaky ferroelectric sample. The increased leakage current, which is ascribed to agglomeration as-synthesized nanoparticles in the composite,^27^ leads to substantial overestimation of the coupling coefficient.^14,28^ It should be noted that the presence of the leakage current also impedes reliable evaluation of the magnetoelectric coupling and leads to an overestimation of the *α*_ME_ value.^14^ We note that distortion of the *D*–*E* loop for the MPCs thin-films with as-synthesized nanoparticles begins at much lower compositions at the loading of 4 wt %. The leakage current problem is much more severe at higher loading of as-synthesized nanoparticles, as shown in Figure S12A.

Good ferroelectric hysteresis loops have been obtained for P(VDF-TrFE) nanocomposites with PMMA-grafted nanoparticles. A representative *D*–*E* displacement loop for the composite with 20 wt % PMMA-grafted nanoparticles is given in Figure 4A, which shows a reduced *P*_r_ (3.1 μC m^–2^) and an increased *E*_C_ (65 MV m^–1^). Since *P*_r_ stems from the crystalline phase of the polymer, a theoretical upper limit of 3.8 μC m^–2^ is expected because the crystallinity of the P(VDF-TrFE) phase is reduced by 65% of its pristine state (see Figure 3B). Obtaining an experimental value similar to the expected theoretical value confirms the internal consistency of the measurements. We note that similar high-quality *D*–*E* loops, albeit with reduced *P*_r_, have been obtained for all nanocomposite thin films with loading as high as 40 wt %, as presented in Figure S12B. The reduction trend in *P*_r_ as a function of PMMA-grafted nanoparticle weight fraction is presented in Figure 4C. Remanent polarization drops monotonically with the weight fraction within the investigated composition range, in agreement with the reported literature.^25^

Next, the values for *d*_33_ for various composites have been estimated. It has been shown that the piezoelectric coefficient, *d*_33_, depends on *P*_r_, *ε*_r_ (the relative permittivity), and *Q*_33_ (the electrostriction coefficient) following *d*_33_ = *d*_coupling_ + 2*Q*_33_*ε*_0_*ε*_r_*P*_r_, where *d*_coupling_ determines the coupling of the crystalline with the amorphous phase, and *ε*_0_ is the permittivity of the vacuum.^31^ The values for *d*_coupling_ and *Q*_33_ for P(VDF-TrFE) amount to −20 pm V^–1^ and −1.5 m^4^ C^–2^, respectively, which vary linearly with increasing *P*_r_, with *d*_coupling_ exhibiting an increasing trend and *Q*_33_ showing a decreasing trend.^31^ Values for *P*_r_ of various composites are given in Figure 4C. To determine the *d*_33_ of the composites, *ε*_r_ has been measured (Figure S14a). The *d*_33_ of the composite has been determined and is given in Figure S14b. The *d*_33_ of the composite decreases almost linearly as the weight fraction of PMMA-grafted nanoparticles increases.

Next, the magnetic properties of the nanocomposites are evaluated. A representative magnetization loop for the nanocomposite with 20 wt % PMMA-grafted nanoparticles, presented in Figure 4B, demonstrates that the composite thin-film exhibits remanent magnetization at room temperature. All nanocomposites exhibit room-temperature saturation magnetization (Figure S12C), with *M*_s_ that increases linearly as the weight fraction of PMMA-grafted nanoparticles increases, as presented in Figure 4C. Therefore, P(VDF-TrFE) nanocomposites thin-films with PMMA-grafted nanoparticles are simultaneously ferroelectric and ferromagnetic; hence room-temperature multiferroic nanocomposite thin-films.

Finally, the out-of-plane *α*_ME_ values for the composite are evaluated. To avoid reporting artifacts, only devices that showed a proper ferroelectric D-E loop, *i*.*e*., no leakage current, are evaluated. Since magnetoelectric devices operated under AC mode, the AC conductivity of the sample should be low. To that end, the dielectric loss, tan(δ) at 1 kHz for samples with different composition of PMMA-grafted nanoparticles is measured, as presented in Figure S13. AC conductivity of the samples with PMMA-grafted nanoparticle remains low, comparable to that of P(VDF-TrFE) neat sample. The tan(δ) slightly increases in conjunction with the reduced permittivity of the composites. Therefore, the AC conductivity of the MPCs remains unchanged and comparable to that of the pristine P(VDF-TrFE) sample (see the Supporting Information for more discussion). Before measurement of the coupling coefficient, an electric field of 70 MV/m larger than the coercive field of P(VDF-TrFE) is applied to set the polarization state of the device to + *P*_r_. Subsequently, a magnetic field larger than the coercive magnetic field of 500 Oe (Figure 1E) has been applied to set a known magnetization state in the nanocomposites. The nanocomposites generate a voltage when placed in an external variable magnetic field, *H*_AC_. As indicated earlier, the magnetically induced voltage, *V*_ME_, on the capacitor electrodes is *V*_ME_ = *α*_ME_·*H*_AC_·*t*, where *t* is the thickness of the nanocomposite layer. The *H*_AC_ is the amplitude of the externally applied magnetic field, which varies from 1 to 9 Oe. The frequency of the applied *H*_AC_ varied from 1 to 10 kHz. A well-established lock-in technique is used to measure magnetically induced voltage, *V*, on the capacitor plates. The schematic of the measurement setup is presented in Figure S15.^28^ Only, the real component of the lock-in signal is reported as the true magnetoelectric signal because the imaginary component is due to the magnetic induction (see Figure S16 and the accompanying explanation). Details of the measurement protocol are explained elsewhere.^28^ First, the benchmark nanocomposites with as-synthesized oleate-coated nanoparticles are measured. Devices with a loading higher than 4 wt % suffer from leakage current, as shown in Figure 4a, which impedes a reliable evaluation of *α*_ME_. The composite with 4 wt % as-synthesized oleate-coated nanoparticles could be reliably evaluated. The *α*_ME_ value amounts to 30 ± 10 mV Oe^–1^ cm^–1^ (at *H*_DC_ = 0.5 T and *H*_AC_ = 0.8 mT at 10 kHz), shown in Figure 4d with a red symbol. The measured *α*_ME_ value for the composite with as-synthesized oleate-coated nanoparticles is comparable to the values reported in the literature for similar systems.^12,29,30^ Next, the nanocomposites with PMMA-grafted nanoparticles are measured. The composite with 5 wt % loading of PMMA-grafted nanoparticles exhibits an *α*_ME_value as high as 450 ± 50 mV Oe^–1^ cm^–1^ (*H*_DC_ = 0 T and *H*_AC_ = 0.8 mT at 10 kHz), as presented in Figure 4D (blue symbols). With increasing the weight fraction of the PMMA-grafted nanoparticles to 20 wt %, the room-temperature *α*_ME_ value of the nanocomposite reaches 750 ± 30 mV Oe^–1^ cm^–1^ (*H*_DC_ = 0 T and *H*_AC_ = 0.8 mT at 10 kHz) and shows a significant increase compared to reported values in the literature presented in Table S1. The grafted nanoparticles reside in the amorphous phase of the matrix, reducing the free volume, thereby increasing Young’s modulus of the P(VDF-TrFE) phase, which in turn yields the generation of a larger voltage upon exerting the same strain to the piezoelectric phase.

Further increase of the PMMA-grafted nanoparticle loading to 36 wt % reduces the *α*_ME_value. The reduction in *α*_ME_ is attributed to the reduced crystallinity and weakening of the piezoelectric response in the P(VDF-TrFE) phase at a high nanoparticle loading, which is also reflected in the reduced *P*_r_ values for these composites.

Figure 4D reveals that, for cobalt ferrite nanoparticles, Co_0.7_Fe_2.3_O_4_, with the size of 13 nm and PMMA shell with a molecular weight of 41 kg mol^–1^, the experimentally determined optimal composition that yields the largest *α*_ME_ is at 20 wt % loadings. Due to its low/medium molecular weight, the PMMA chains do not coil or entangle but stretch outward from the nanoparticle’s surface. The PMMA and P(VDF-TrFE) strongly interact through electronic dipolar interactions and are locked up together. Hence, PMMA shells do not obstruct strain transfer. Tentatively, for high molecular weight PMMA shells, coiling of the chains is expected. The coiled PMMA region primarily interacts with the P(VDF-TrFE) matrix, adversely affecting the strain transfer. The molecular weight of the PMMA shell used in our experiments, 41 kg/mol, is considered low/medium. Hence, efficient strain transfer is guaranteed. Furthermore, it should be noted that the weight fractions refer to the weight of the PMMA-grafted nanoparticles, not that of PMMA. The weight of PMMA is always much less than that of the grafted nanoparticle because of the significant difference in the mass density between cobalt ferrite (∼ 6 gr/cm^3^) and PMMA (∼ 1.2 gr/cm^3^).

The coupling coefficient values in Figure 4D have been obtained in the absence of an external DC magnetic field, *H*_DC_. To confirm that the composites are self-biased, the coupling coefficient for the composite with the most significant response, *i*.*e*., the one with 20 wt % loading of PMMA-grafted nanoparticle, is measured. As shown in Figure S17a, similar *α*_ME_ values are obtained at various *H*_DC_s, confirming that the nanocomposite with PMMA-grafted nanoparticles is self-biased. The dependence of the coupling values on the frequency and amplitude of *H*_AC_ is reported in Figure S17b. The *α*_ME_ increases with increasing *H*_AC_ amplitude and shows a saturating behavior for the probed frequency range.

## Conclusion

In summary, thin films of polymer-based nanocomposites with large magnetoelectric coupling coefficients have been demonstrated by preventing agglomeration of the magnetic nanoparticle. By maximizing the interface between the nanoparticles and the piezoelectric matrix through nanoparticle size reduction, it is finally possible to experimentally demonstrate solution-processed thin-film nanocomposites that show a coupling coefficient of 750 ± 30 mV Oe^–1^ cm^–1^. Since the nanoparticles are magnetic at room temperature, their resulting composite shows magnetoelectric coupling even in the absence of an external DC magnetic field. The breakthrough in uniform nanoparticle dispersion in thin films is achieved by mitigating the agglomeration through surface-initiated polymerization of poly(methyl methacrylate) (PMMA) chains grafted on the surface of the cobalt-ferrite nanoparticles, which renders the nanoparticles miscible in a P(VDF-TrFE) matrix. An important note is that the large value reported here is by no means the upper limit because there are experimentally many parameters that can be explored, such as the use of highly magnetostrictive nanoparticles, to enhance the coupling response of the system even further. Demonstrating solution-processed multiferroic films with submicrometer thicknesses and a giant magnetoelectric coupling coefficient is a significant advancement in the field of multiferroic thin-films and enables realization of various (flexible) multiferroic electronic devices, for example, in battery-free, remotely powered wearables and implantable sensors for health monitoring,^36^ in harvesting devices^37^ that harvest ambient and stray electromagnetic fields, or in accurate magnetoelectric sensors.^38^ The methodology employed here is generic and can be adapted to various polymer nanocomposite systems, where the Holy Grail is to achieve a uniform filler dispersion in a polymer matrix.

## Methods

### Materials

Iron(III) acetylacetonate (99.99%), cobalt(II) acetylacetonate (99.99%), oleylamine (OAM, > 70%), benzyl ether (BE, technical grade 99%), oleic acid (OAC, technical grade, 90%), 4,4′-dinonyl-2,2′-bipyridine (dNbipy, 97%), copper(I) bromide (Cu(I)Br, 99.9%), *p*-toluene sulfonyl chloride (TsCl, 99%), methyl methacrylate (MMA, 99%), hydrochloric acid (HCl), nitric acid, extra dry toluene (99.99%), extra dry diethyl ether (99.99%), hexane, ethanol, tetrahydrofuran, and acetone were purchased from Sigma-Aldrich. 1,2-Hexadecandiol (99%) was purchased from TCI. 2-(4-chlorosulfonylphenyl)ethyltrichlorosilane (CTCS, 50% solution in CH_2_Cl_2_) was purchased from ABCR. P(VDF-TrFE) (65–35%) was purchased from Solvay and used as received.

### Synthesis

#### Synthesis of Cobalt-Ferrite Nanoparticles of Different Sizes

Cobalt-ferrite nanoparticles were synthesized by the thermal decomposition method (as we described previously).^19,33,34^ Fe(acac)_3_ (2 mmol) with 1.5 mmol of Co(acac)_2_, 1,2-hexadecanediol (10 mmol), oleic acid (6 mmol), oleylamine (6 mmol), and benzyl ether (20 mL) were mixed and magnetically stirred under a flow of nitrogen. The mixture was heated to 110 °C and stayed at that temperature for 60 min under a vacuum. Then, the mixture was heated to 180 °C with a heating rate of 6.5 °C/min and remained at that temperature for 2 h. Under nitrogen purging, the solution was heated to reflux temperature (∼ 295 °C) with a heating rate of 3.3 °C/min and was kept at reflux for 60 min. The black mixture was then cooled to room temperature and washed three times with a mixture of toluene/ethanol/acetone followed by centrifugation (6000 rpm, 10 min) and finally stored under argon in toluene or hexane.

#### Surface Initiation of Cobalt-Ferrite Nanoparticles

As-synthesized nanoparticles were covered with oleate.^32^ To initiate the surface of the nanoparticles, we exchanged oleate surfactant from synthesis with CTCS.^24,34,35^ To do so, the nanoparticles were dried at 60 °C under a vacuum overnight. Then, 100 mg of nanoparticle was put in a two-necked degassed flask (degassed three times and refill with argon), followed by adding 30 mL of extra dry toluene under argon blanketing. The mixture was kept at 25 °C during the exchange, under bath sonication (equipped with temperature controller, 3510 Branson). Sonication was used to keep the nanoparticles dispersed in toluene during the exchange reaction and later to obtain a homogeneous CTCS coverage. After 20 min, CTCS was added dropwise while sonicating. Different parameters, such as CTCS concentration and reaction time, were investigated. The optimized conditions for the full exchange were 3 mmol of CTCS per gram of nanoparticle and a reaction time of 3 h. The exchange takes place through the condensation reaction of silane molecules on the cobalt-ferrite surface. FTIR spectra (Figure 1D) confirms full ligand exchange as the carbonyl and CH_2_ bands of the oleate disappear, and S=O, Si—O—Si, and Fe—O—Si bands of CTCS emerge. The modified MNPs were washed four times with fresh THF and two times with toluene by consecutive separation and redispersion. Finally, the MNPs were dispersed in toluene and stored under argon.

#### SI-ATRP of PMMA Brushes

MMA was purified by washing two times with 5% NaOH solution to remove the inhibitor, followed by washing two times with deionized water and then drying over anhydrous MgSO_4_. Afterward, MMA was distilled over CaH_2_ and then degassed by argon bubbling and stored under an argon atmosphere in the fridge (−20 °C). Copper bromide was purified with glacial acetic acid (five times), washed with pure ethanol (three times), then washed with extra dry diethyl ether (five times), and stored under argon. Other chemicals were used as received.

The initiator-fixated nanoparticles were redispersed in dry toluene (8.3 wt %) in a Pyrex glass tube. Subsequently, MMA (32 g), TsCl (19 mg), and dNbipy (485 mg) were quickly added. TsCl and dNbipy were used as free initiators and ligands for complexation with copper, respectively. After Cu(I)Br (85.2 mg) was added to the mixture, the glass tube was immediately degassed four times by freeze–pump–thaw cycles and sealed off under a vacuum. The polymerization was carried out in a temperature-controlled bath sonication and in the presence of a sacrificial (free) initiator, TsCl, to control the so-called process of persistent radical effect.^23,36^ The polymerization solution was constantly sonicated to avoid aggregation during the polymerization of the nanoparticles. The reaction temperature was 50 °C. The reaction was performed on different time scales, after which the solution was quenched to room temperature. The reaction mixture was diluted with acetone and centrifuged (25000 rpm, 3 h) to gain the polymer-grafted magnetic nanoparticles. The centrifugation and redispersion cycle was performed five times to collect PMMA-grafted nanoparticles without any trace of free, unbounded PMMA chains. To determine the molecular weight, PMMA was cleaved from the nanoparticles by adding 2 mL of 37% aqueous HCl to 20 mg of PMMA-grafted nanoparticles dispersed in toluene. The mixture was vigorously stirred for 2 h. The organic part of the solution was washed with aqueous NaHCO_3_ solution and water, precipitated in methanol, filtered, and then subjected to GPC. We note that the molecular weight of the free polymer (in the reaction solution) and the grafted polymer on the surface were the same. The molecular weight of the PMMA shell amounts to *M*_n_ = 41000 g/mol. The polydispersity index, PDI, of the grafted-PMMA is below 1.2, indicating that the polymerization proceeded in a controlled living fashion.

### Thin-Film Capacitor Fabrication

Composite thin films were prepared by dissolving various amounts of the PMMA-modified nanoparticles with P(VDF-TrFE) in a common organic solvent. Thin films were realized by spin-coating or bar coating under low humidity conditions (below 10%) to suppress vapor-induced phase separation (VIPS)^25,32^ and to obtain compact thin films. To prepare capacitors, glass slides were first thoroughly cleaned in acetone, propanol, and DI water. As the bottom electrode, 50 nm Au/1 nm Cr electrodes were evaporated. Films of P(VDF-TrFE) or its composites were formed by spin coating under low humidity of <10% to suppress VIPS.

### Physical characterizations

#### Transmission Electron Microscopy

TEM images (taken using JEOL JEM1400) were used to characterize the nanoparticle size and distributions. The size distributions were obtained from statistical size analysis of more than 2000 particles using ImageJ software by assuming spherical particles. The average particle size and distribution width were obtained by fitting Gaussian distribution functions to the particle size histograms.

#### X-ray Diffraction

XRD studies were performed at room temperature using a diffractometer equipped with a monochromatic copper radiation source CuKα (λ = 1.5406 Å) in the 15–65° (2θ) range with a scan step of 0.03°. The mean size and lattice parameter of the crystal domains for the nanoparticles were calculated from the XRD pattern by using the Scherrer and Bragg equations.

#### Gel Permeation Chromatography

GPC measurements were carried out on an Agilent Technologies 1260 Infinity system equipped with a RI and a UV detector. THF was used as solvent with a flow rate of 1 mg/mL and with PMMA as the standard reference.

#### Fourier Transform Infrared Spectroscopy

FTIR spectra of the samples were recorded between 400 and 4000 cm^–1^ with a PerkinElmer FTIR spectrometer. To obtain the spectra, the nanoparticles were gently ground and diluted with KBr and compressed into a pellet.

#### Thermogravimetric Analysis

TGA was performed on powder samples that were dried at 80 °C in a vacuum oven overnight. The temperature was increased from 20 to 800 °C at 10 °C/min under N_2_.

#### Inductively Coupled Plasma Optical Emission Spectroscopy

ICP-OES analysis (ACTIVA M) was performed to quantify the cobalt and iron concentration. The nanoparticles were digested in concentrated aqua regia (3:1 ratio of hydrochloric acid to nitric acid) for 1 h. The resulting solutions were diluted to 50 ppm. The metal calibration standards (0.1–100 ppm) were prepared by diluting aliquots from Inorganic Ventures stock solutions of 1000 ppm metal content (iron, cobalt). Considering Co_*x*_Fe_3–*x*_O_4_ as the composition for the cobalt-ferrite nanoparticle, the cobalt stoichiometry (*x*) was then calculated according to the following expression:^19^
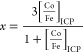


#### Multiferroic Measurements

The magnetic properties of the nanoparticles and PMMA-modified ones were measured using a VSM (Cryogenic Ltd.) magnetometer. The magnetization, *M*–*H*, loops were measured under a maximum applied field of 50 kOe at 300 K. The magnetization versus temperature measurements were performed in zero-field-cooled (ZFC) and field-cooled (FC) conditions with a 100 Oe probe field. The ferroelectric properties were measured using the Sawyer–Tower circuit. The magnetically induced voltage coefficient was determined using a lock-in technique, as described elsewhere.^28^

## Supporting Information Available

Discussions of agglomeration versus uniform dispersion, finite-element simulations, uniformly dispersed versus agglomerated nanoparticles in the polymer matrix, kinetic of surface-initiated polymerization, and calculation of the graft density, figures of evolution of the interfacial area with the degree of agglomeration, example of defining PMC geometry for finite-element method simulation, definition of the simulation geometry, typical examples of simulated composites with different nanoparticle dispersion, kinetic of surface-initiated polymerization, PMMA-grafted cobalt-ferrite nanoparticle, TGA and XRD of the nanoparticles, TEM images of PMMA-grafted cobalt ferrite nanoparticles, center-to-center distance between PMMA-grated cobalt ferrite nanoparticles as a function of the degree of polymerization of the grafted PMMA, DSC thermogram for P(VDF-TrFE) nanocomposites, SEM microstructure of pristine P(VDF-TrFE), polarization and magnetization characterization of the nanocomposite thin films, dielectric permittivity and loss for composites of P(VDF-TrFE) with PPMA-grafted cobalt ferrite nanoparticles as a function of composition, measured dielectric constant and remanent polarization of the composite, estimated *d*_33_ for various composite based on the measured values, schematic of the setup for measuring magnetoelectric coupling, different components of the lock-in amplifier signals, and frequency, *H*_AC_, and *H*_DC_ dependence of α_ME_, and table of overview of the magnetoelectronic (ME) coefficient of different polymer-based multiferroic composites (PDF)
