# Supplementary material for: Solution-Processed Multiferroic Thin-Films with Large Magnetoelectric Coupling at Room-Temperature
Source: ACS Nano. 2023 Apr 17;17(9):8064–73. doi: 10.1021/acsnano.2c09769 (PMC10173693; doi:10.1021/acsnano.2c09769)
Supplement: Supplementary file 1 — nn2c09769_si_001.pdf [file nn2c09769_si_001.pdf]

Supporting Information

Solution-Processed Multiferroic Thin-Films with Large Magnetoelectric Coupling at Room-Temperature

*Hamed Sharifi Dehsari[1†], Morteza Hassanpour Amiri[1†], Kamal Asadi[1,2,3]**

1 Max Planck Institute for Polymer Research, Ackermannweg 10, 55128, Mainz, Germany

2 Center for Therapeutic innovations, University of Bath, Bath, United Kingdom.

3 Department of Physics, University of Bath, Claverton Down, BA2 7AY, Bath, United Kingdom.

* Corresponding author: Email: ka787@bath.ac.uk

† These authors contributed equally to this work.

This PDF file includes:

Supplementary Text
Figures. S1 to S14
Tables S1

Agglomeration versus uniform dispersion

The ME coupling in polymer composites stems from interfacial interactions and strain transfer. Agglomeration of the nanoparticles severely reduces the interfacial area and strain transfer, reducing the ME coupling. To demonstrate the effect of agglomeration, we calculated the total interface area for the case of individually dispersed non-agglomerated nanoparticles and compared that with composites with various degrees of agglomeration and plotted the results in Figure R2. The following assumptions are made for this calculation: the composite with 20wt% nanoparticles is considered, with a mass density of ~ 6 $gr.cm^{-3}$ and a diameter of 13 nm (the same as the particles used in our work) have been assumed. For a fully dispersed composite, every cluster of nanoparticles contains only one particle. The total interfacial area is 15.4 m^2 per gram of the composite. Next, different degrees of agglomerations are considered by clustering 10 (or more) nanoparticles together, assuming (for simplicity) that clusters also adopt a spherical shape. The calculated total interfacial area shows a sharp drop to 1.8 m^2 per gram of the composite for clustering 10 nanoparticles, which can be considered a minimal degree of agglomeration. Further increase in cluster size reduces the total interfacial area even further. For clusters of 10000 nanoparticles, the interfacial area is reduced by nearly two orders of magnitude to just 0.17 m^2 per gram of composite.

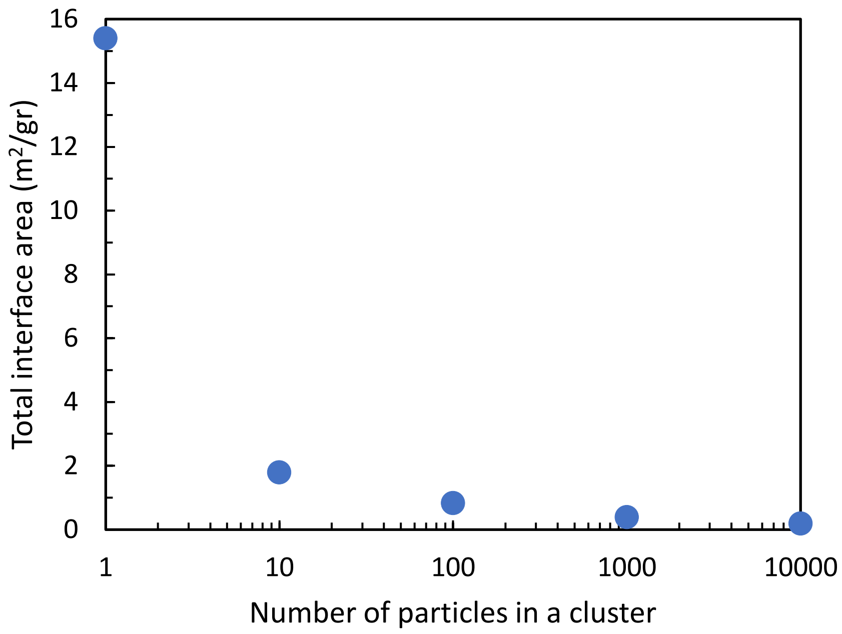

Figure S1. Evolution of the interfacial area with the degree of agglomeration. The degree of agglomeration has been defined as the number of nanoparticles in a nanoparticle cluster.

Finite-element simulations

Finite-element simulations of multiferroic composite have been performed Using COMSOL. To that end, three modules related to the electromagnetic, piezoelectric, and mechanical properties have been employed. Hence, the program solves Maxwell's equations at every grid point. The mechanical properties of the composite are calculated using the differential form of Newton's second law (Eq. 1) and strain displacement equation (Eq. 2):

$$\rho \frac{\partial^2 u}{\partial t^2} = \nabla . s + F_V \qquad (1)$$

$$\epsilon = \frac{1}{2}(\nabla u)^T + \nabla u. \qquad (2)$$

Where u is displacement, ρ is the material's density, s is stress, F_V is volumetric force, and ϵ is strain. The Maxwell equations and the mechanical properties are coupled through the piezoelectric properties of the composite following:

$$S = s_E T + d^T E \qquad (3)$$

$$D = \varepsilon_0 \varepsilon_{rT} E + dT \qquad (4)$$

Where S is strain, T is stress, $\varepsilon_0$is vacuum permittivity, s_E, ε_{rT} and d are compliance, relative permittivity, and piezoelectric charge coefficient tensors. Equations 3 and 4 describe strain-charge coupling.

To simulate the composite microstructure, spherical nanoparticles are assumed, as in Figure S1a. For the case of the PMMA-grafted nanoparticles, the surface of the particles is covered with an elastic non-magnetic shell with Young's modulus that is similar to that of PMMA, 2.9GPa, as shown in Figure S1b. A P(VDF-TrFE) cube is defined wherein the nanoparticles are randomly distributed, Figure S1c. For the P(VDF-TrFE) matrix, Young modulus and piezoelectric voltage coefficient of 2.5 GPa and -0.372 V m N^{-1} are used, respectively. It should be noted that the position of the nanoparticles is not fixed between different simulation runs and is fully randomized. The only constraint that is imposed is that the nanoparticles do not infuse. The following parameters were used for the piezoelectric phase:

Relative permittivity:

$$\varepsilon_{rT} = \begin{pmatrix} 7.4 & 0 & 0 \\ 0 & 9.3 & 0 \\ 0 & 0 & 7.6 \end{pmatrix}$$

Compliance:

$$s_E = \begin{pmatrix} 3.781 & -1.482 & -1.724 & 0 & 0 & 0 \\ -1.482 & 3.781 & -1.724 & 0 & 0 & 0 \\ -1.724 & -1.724 & 10.92 & 0 & 0 & 0 \\ 0 & 0 & 0 & 14.28 & 0 & 0 \\ 0 & 0 & 0 & 0 & 11.1 & 0 \\ 0 & 0 & 0 & 0 & 0 & 11.1 \end{pmatrix} \times 10^{-10}\ \mathrm{Pa}^{-1}.$$

Piezoelectric coupling:

$$d = \begin{pmatrix} 0 & 0 & 0 & 0 & 0 & 0 \\ 0 & 0 & 0 & 0 & 0 & 0 \\ 13.58 & 1.476 & -33.8 & 0 & 0 & 0 \end{pmatrix} \times 10^{-12}\ \mathrm{CN}^{-1}.$$

Density: 1780 kg/m^3.

The electrical potential is calculated by placing two gold electrodes on the cube's top and bottom (along the z-direction). The construct is then placed in an air cube, Figure S1c (or sphere, as will be discussed later), to evaluate the magnetoelectric response of the composite. A voltmeter is connected to the Au electrodes to record the generated V_{AC} as a function of the time-varying magnetic field (H_{AC}). To obtain the multiferroic response, a uniform, time-varying H_{AC} is required. To that end, a solenid, Figure S2a, is defined to create a uniform time-varying magnetic field at its

center using an AC. An air sphere is defined around the solenoid, wherein the field is calculated. A snapshot of the magnetic field is shown in Figure S2b. The used geometry generates a uniform, time-varying magnetic field. At last, the PMCs, as defined in Figure S2c, are placed in the center of the solenoid, as shown in Figure S2c. Subsequently, the current passing through the solenoid is varied, and the action of the resulting magnetic field on the PMC is calculated.

Uniformly dispersed versus agglomerated nanoparticles in the polymer matrix

For a fair comparison, a fixed number of particles is used in both scenarios so that uniform dispersion and agglomeration systems have a similar interfacial area between the nanoparticles and the piezoelectric matrix. For every case, at least 10 different randomly generated microstructures are calculated to reach reliable statistics. Figure S3 shows a few examples of both uniform and agglomerated cases. From the magnetically induced voltage, the values for α_{ME} for every structure is calculated. For the sake of comparison, we have normalized all the calculated α_{ME} by dividing them by the largest α_{ME}Which is obtained for the case with uniform nanoparticle dispersion. The normalized α_{ME}s for every case are averaged and presented in Figure 1d of the main text. It should also be noted that in the simulation, the whole volume of the matrix is considered piezoelectric, even the volume enclosed by the agglomerated particles. This limitation of the software becomes advantageous in better understanding the experimental results. Our code defines the nanoparticles such that they do not merge upon agglomeration. The interface between the nanoparticle and the piezoelectric phase for uniform dispersion and agglomeration cases remains practically the same.

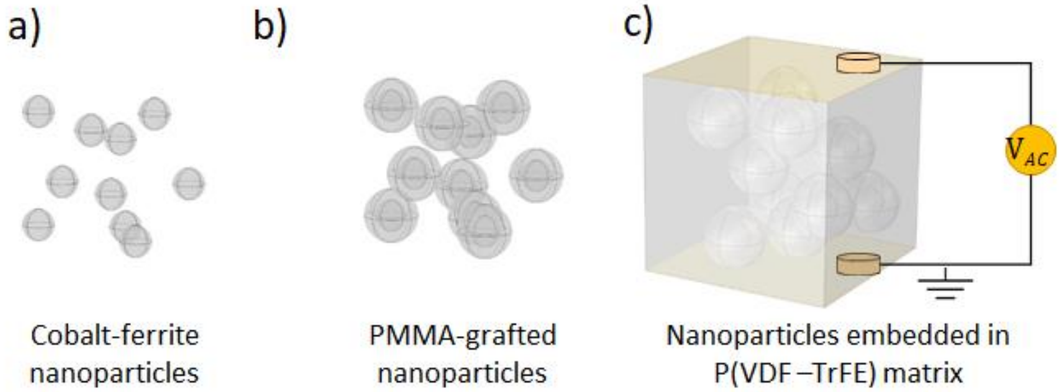

Figure S2. An example of defining PMC geometry for finite-element method simulation. (a) bare nanoparticles, (b) PMMA-grafted nanoparticles, and (c) the same nanoparticles in (b) that are embedded in a P(VDF-TrFE) matrix, which is contacted with top and bottom Au electrodes. The voltmeter reads V_{AC}, which is generated upon application of H_{AC}.

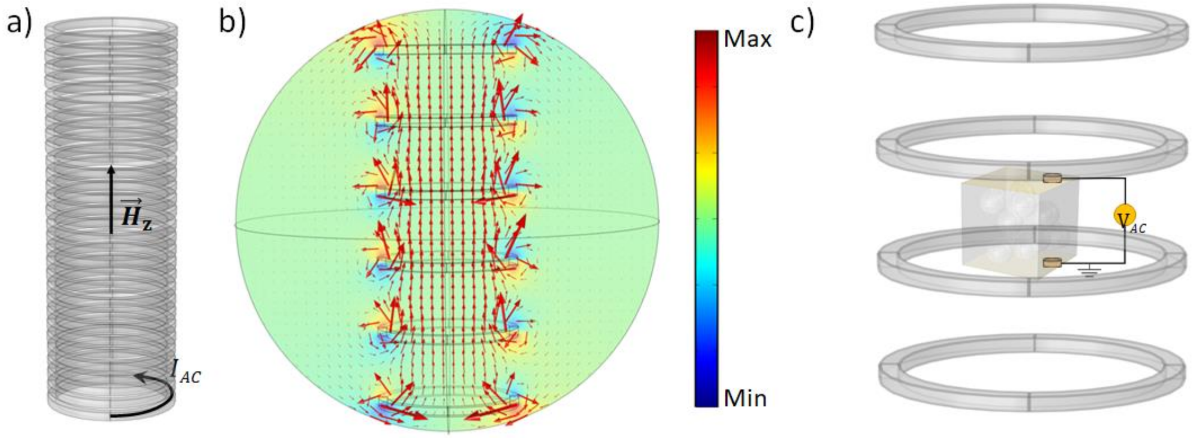

Figure S3. Definition of the simulation geometry. (a) First, a solenoid is defied, wherein an AC current passes. (b) The geometry is optimized to yield a uniform field at the center of the solenoid. (c) The PMC is placed at the center of a solenoid to calculate the magnetoelectric voltage coefficient. (note that some of the solenoid turns are removed for image clarity). The images are not to the scale used for the simulations.

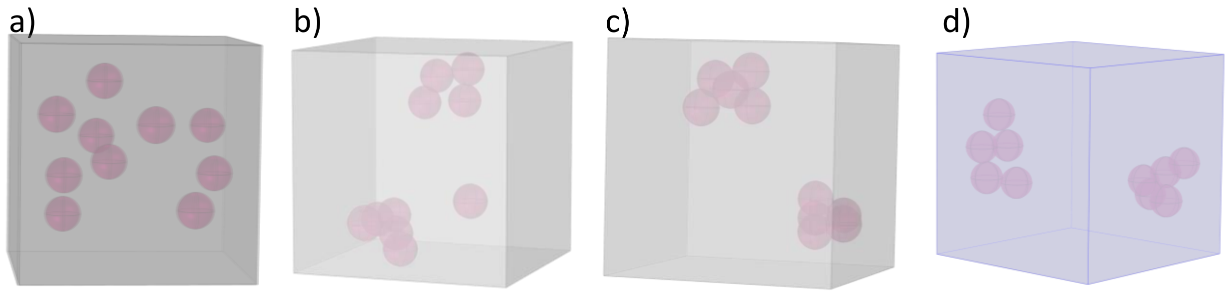

Figure S4. Typical examples of simulated composites with different nanoparticle dispersion. (a) a typical case of a uniform dispersion scenario, and (b-d) examples of agglomeration of the nanoparticles. The nanoparticles are set to be in close contact for agglomeration but not merging. The rest of the cube, including the volume enclosed between the agglomerated nanoparticles, is piezoelectric.

Kinetic of surface-initiated polymerization

Determining the kinetic of SI-ATRP enables a good degree of control over the molecular weight of the PMMA chains. The kinetic of SI-ATRP is determined by monitoring the MMA monomer consumption over time. The change in monomer concentration during the polymerization, f0, is linear in time. Hence the polymerization follows first-order kinetic.[1,2] As shown in Figure S1b, M_n grows linearly as a function of conversion. After cleavage from the nanoparticle surface, we compared the molecular weight of the grafted PMMA with that of a free polymer. As illustrated in Figure S1b, M_n values of the grafted-PMMA (hollow symbols) and a free polymer (filled symbols) are nearly identical, and both increase via increasing the monomer conversion. Hence, both the free and grafted polymers have similar growth kinetics.

Following the conditions for a living polymerization, where all propagating chains grow at the same rate and for the same length of time, the molecular weight of polymerization can be calculated by:

$$M_{n,theory} = \frac{M_{MMA} \times C \times MMA._0}{TsCl._0 + CTCS\ on\ surface._0} \qquad (5)$$

where M_{MMA} is the molecular weight of MMA, C is the conversion, and $MMA._0$ and $TsCl._0$ are the concentrations of MMA and free initiator in the feed, respectively. As we discussed earlier, the initiation sites available on the MNPs surface by assuming the spherical shape were calculated from the CTCS grafting density. The M_n values (black squares) agree well with $M_{\text{n-theory}}$ (dotted line), proving that the reaction follows the first-order kinetic. In addition, the PDI (defined as the ratio of molecular weight to number-average molecular weight, *i.e.*, M_w/M_n) of the grafted PMMA shell remained almost below 1.2, proof that the graft polymerization proceeded in a living fashion.[3,4]

TGA tests were also performed for PMMA-grafted cobalt ferrite nanoparticles to determine the grafting density of PMMA on the surface of magnetic nanoparticles. The grafting density of PMMA on the surface reaches 0.4 chains per nm^2. We also noticed that the grafting density remains nearly unchanged and independent of polymerization time and the molecular weight, which indicates the growth of initially grafted chains by increasing reaction time.

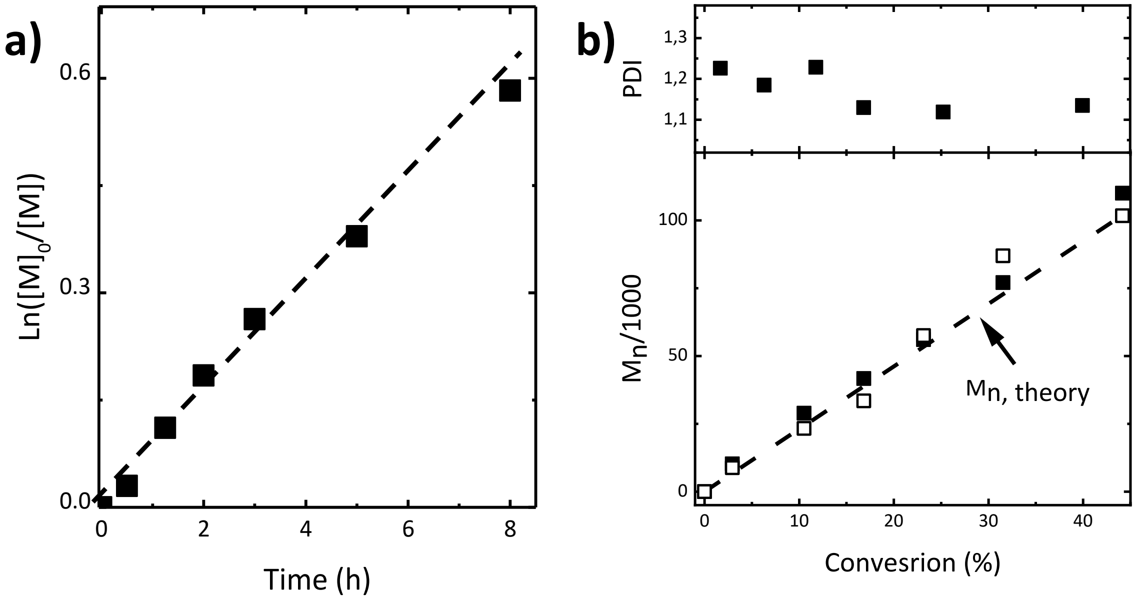

Figure S5. Kinetic of surface-initiated polymerization. (a) The plot of ln(M.0/M.) versus time for MMA polymerization at a reaction temperature of 50 for cobalt ferrite nanoparticles with MMA.0 : p-TsCl.0 : Cu(I)Cl.0 : dNbipy.0 3000: 1: 6: 12, and (b) the evolution of M_n and PDI (top) and M_n (bottom) of free PMMA (filled symbols) and grafted-PMMA (hollow symbols) as a function of monomer conversion (Hence we can assume the similar molecular weight for both free polymer and grafted polymer). The dotted lines in panels (b) show theoretically calculated M_n.

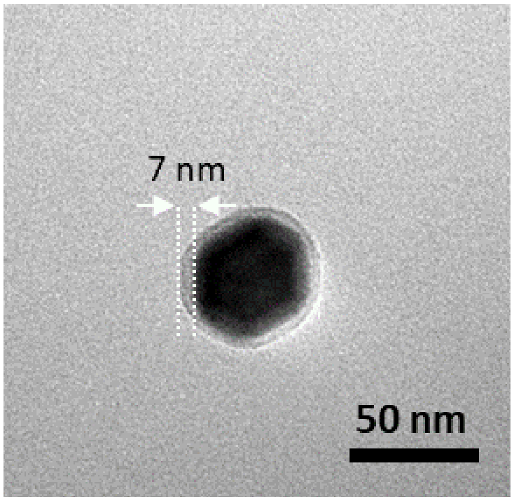

Figure S6. PMMA-grafted cobalt-ferrite nanoparticle. TEM image of a single nanoparticle with a PMMA shell grafted from the surface using ATRP. Note that a larger nanoparticle was used intentionally to produce enough contrast for the PMMA shell to make it visible under TEM.

Calculation of the graft density

The grafting density was calculated from the TGA data (Figure S5a) according to the following equations:

Number of molecules (CTCS or polymer), N: $N = \frac{m_1 - m_3}{M} \times N_A$ (6)

Number of nanoparticles, N_P: $N_P = \frac{m_3}{\rho \times V}$ (7)

Grafting density (molecules nm^{-2}), G.D.: $G.D. = \frac{N}{N_P \times A}$ (8)

Where m_1 and m_3 are the amounts of mass in the first and third DTA peaks, ρ is the density of NPs (~ 5.18 g cm^{-3}), M is the molecular weight of capped surfactant (CTCS or polymer), V is the volume of each nanoparticle, and A is the average area of a single nanoparticle. The CTCS grafting density amounts to 2.7 molecules per nm^{-2}.

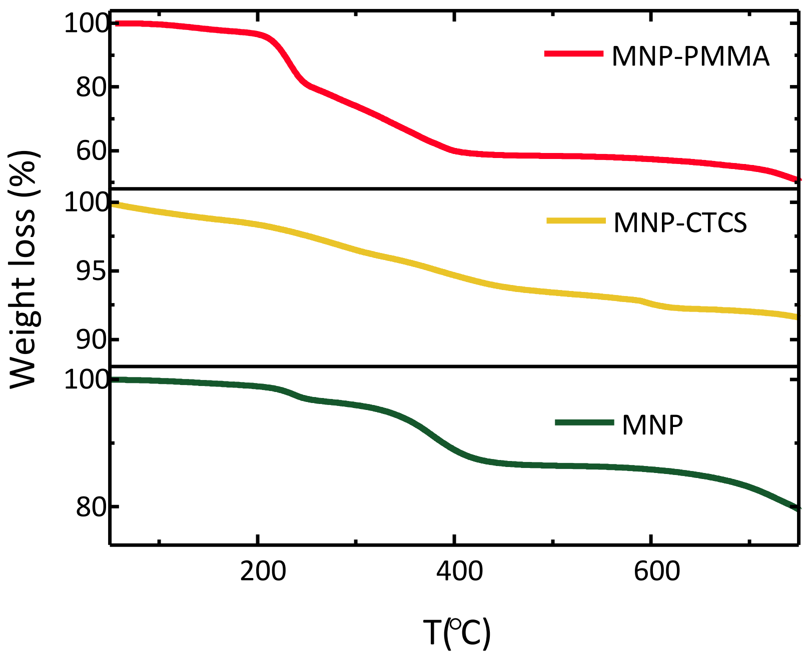

Figure S7. TGA and XRD of the nanoparticles. TGA of the as-synthesized, CTCS-coated, and PMMA-grafted nanoparticles. MNP stands for magnetic nanoparticle.

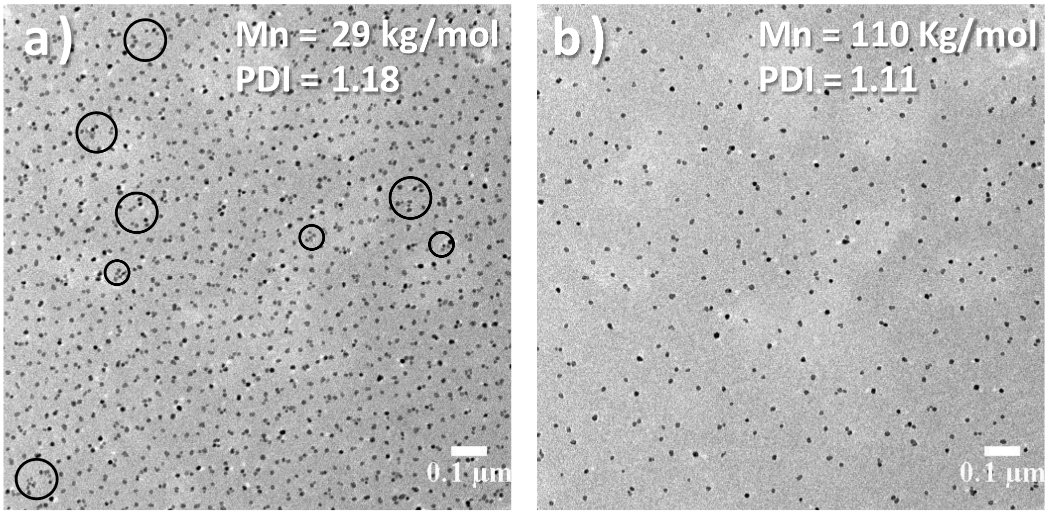

Figure S8. TEM images of PMMA-grafted cobalt ferrite nanoparticles with a PMMA shell of a) low molecular weight and b) high molecular weight

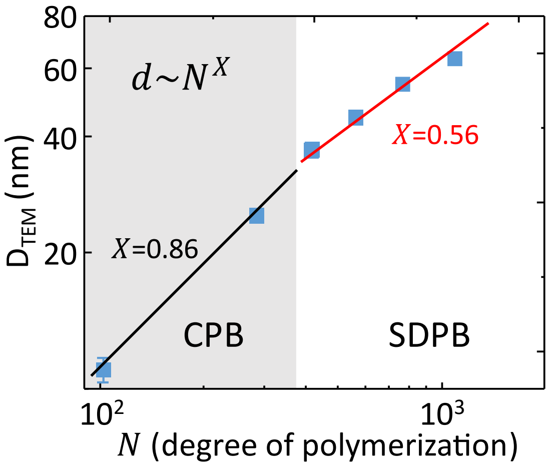

Figure S9. The center-to-center distance between PMMA-grated cobalt ferrite nanoparticles as a function of the degree of polymerization of the grafted PMMA.

Concentrated particle brush (CPB) and semi-dilute particle brush (SDPB) were obtained from the interparticle enter-to-center distance, D_{TEM}, for the PMMA-grafted particles as a function of the degree of polymerization of the grafted PMMA (N). Note that the same cobalt ferrite nanoparticles with diameters of 13.6 nm were used. The enhancement of D_{TEM} as a function of M_n is much more pronounced in lower N than in higher N values. In the CPM regime, due to repulsion between polymer chains, polymer chains are extended, whereas in the SDPB regime, a transition to more relaxed coil conformations of the polymer chains occurs. It has been shown that when the chain conformations in the SDPB regime, the mechanical properties of particle brush are significantly enhanced due to the entanglement of the chains. It has been shown theoretically that CPB and SDPB can be described with a power law, $d{\sim}N^X$, where the exponent X for the CPB regime is $0.6<X<1$ while for the SDPB regime $X{\sim}0.6$.[32-36] We have obtained similar values of X, as depicted in **Figure S9**. A similar trend and values of X have also been reported for silica nanoparticles by Ohno.[37]

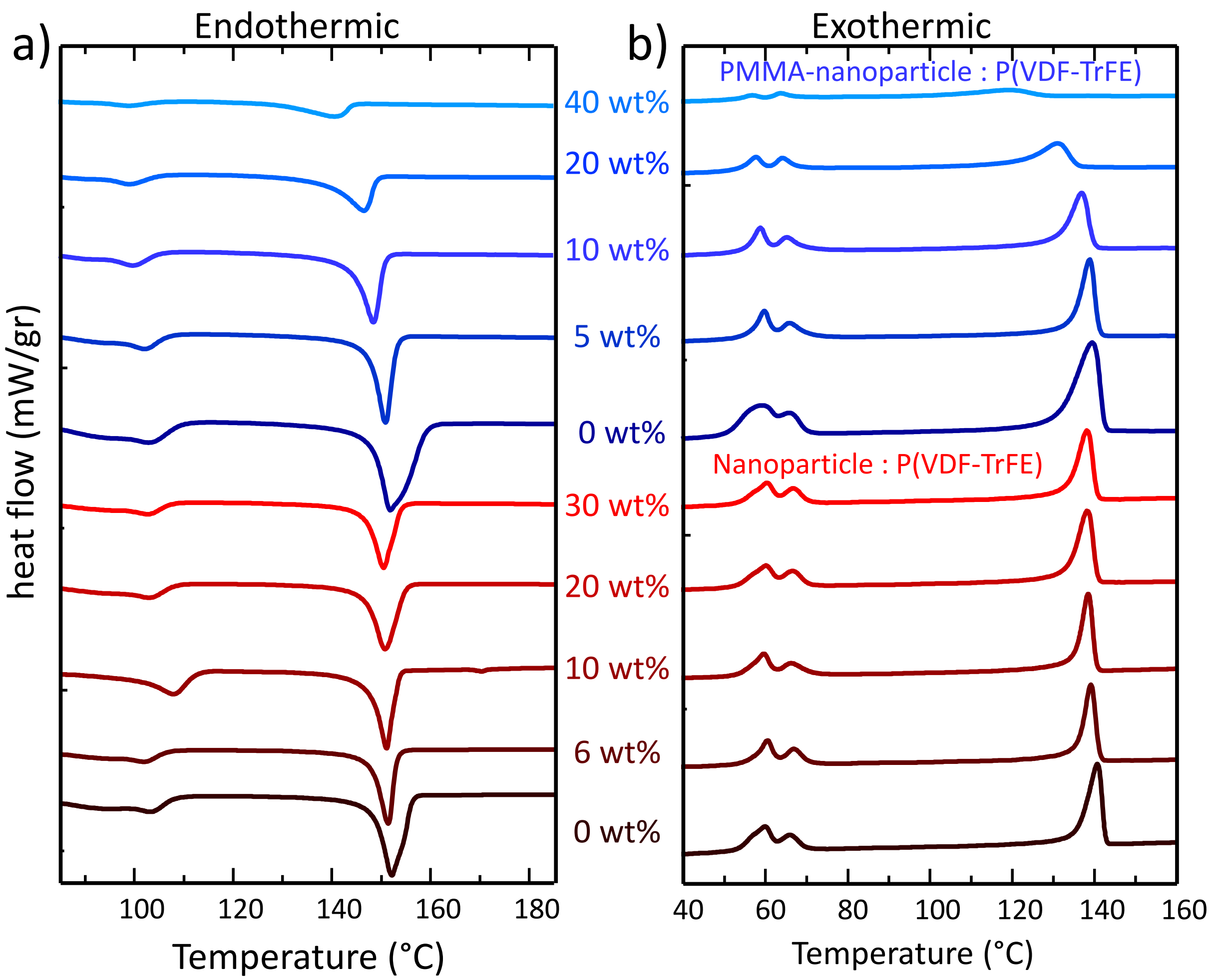

Figure S10. DSC thermogram fo P(VDF-TrFE) nanocomposites. (a) The first heating and (b) cooling curves for the composites with different wt% of as-synthesized nanoparticles (red) and PMMA-grafted nanoparticles (blue)

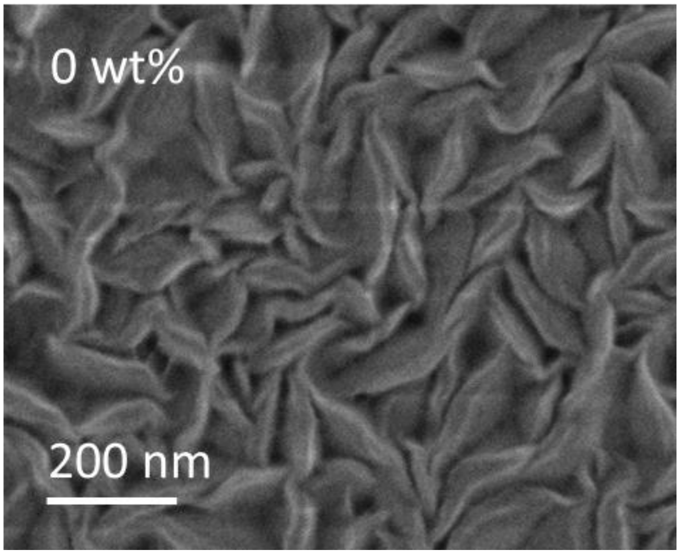

Figure S11. SEM microstructure of pristine P(VDF-TrFE). The needles are P(VDF-TrFE) crystallites randomly distributed in the bulk of the film.

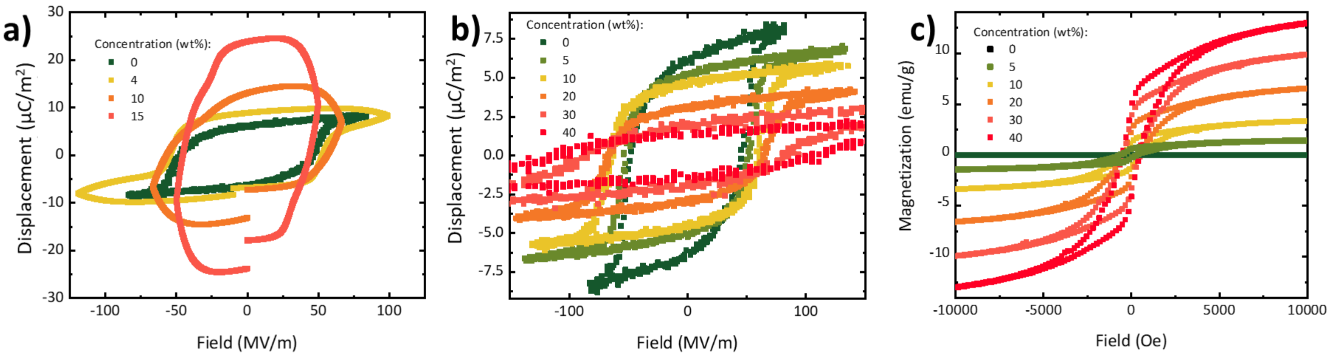

Figure S12. Polarization and magnetization characterization of the nanocomposite thin films. Polarization, *P-E*, response of the nanocomposite thin films of P(VDF-TrFE) with (a) as-synthesized nanoparticles and (b) PMMA-grafted nanoparticles with various loadings. (c) Magnetization, *M-H*, response of the nanocomposite thin films using PMMA-coated nanoparticles at different loadings.

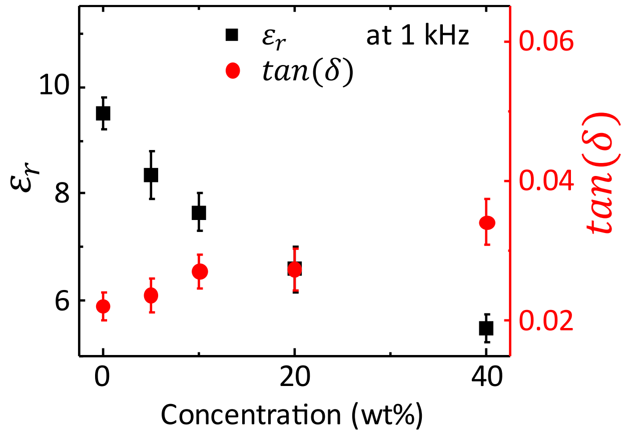

Figure S13. Dielectric permittivity and loss for composites of P(VDF-TrFE) with PPMA-grafted cobalt ferrite nanoparticles as a function of composition.

Dielectric loss, $tan(\delta)$, obtained from the impedance, in combination with relative permittivity is an used as a measure of the AC conductivity of the sample. To evaluate $tan(\delta)$, dielectric permittivity and loss permittivity of the ME composites were measured at 1 kHz. **Figure S13** shows that the relative permittivity drops steadily by a factor of 1.7 from ~9.5 for neat P(VDF-TrFE) to ~5.5 for the composite with 40 wt% PMMA-grafted nanoparticle. Due to this drop in real part of the permittivity, $tan(\delta)$ which is defined as the ration of the dielectric loss to real permittivity shows an slight increase of about a factor of ~1.7 from ~0.02 to 0.035. This means that the dielectric loss for all samples of various compositions remains unchanged. In another word, upon loading of PMMA-grafted nanoparticles the AC conductivity of the sample does not change. Hence the suitability of such composites for device applications.

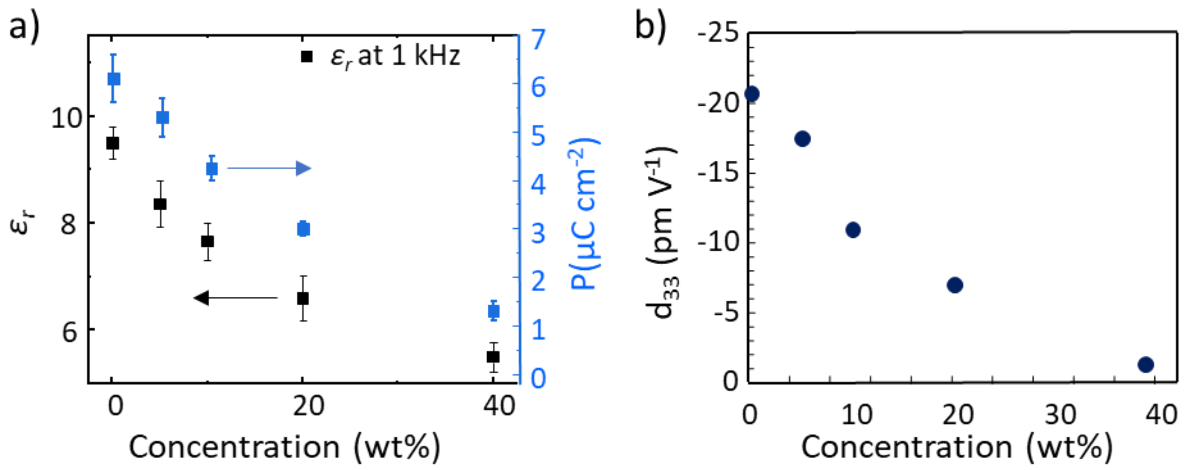

Figure S14. (a) Measured dielectric constant and remanent polarization of the composite at various compositions. (b) Estimated d_{33} for various composite based on the measured values in part a).

The following equations have been used for $Q_{33} = 0.427 \times P_r - 3.459$ and $d_{coupling} = -3.246 \times P_r + 3.174$ (following the work of Katsouras et al.).[5]

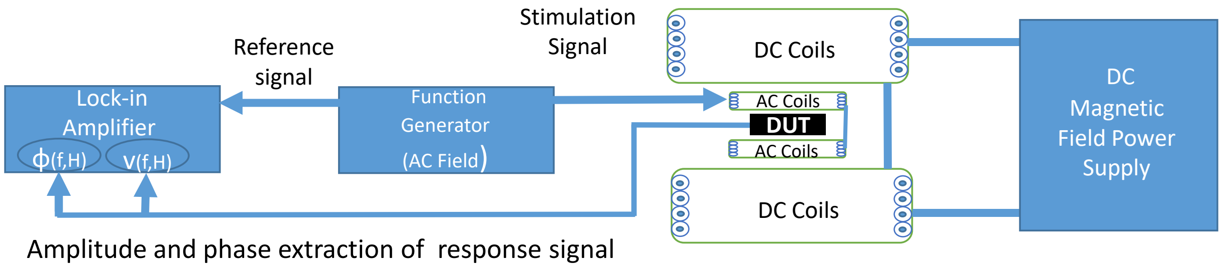

Figure S15. Schematic of the setup for measuring magnetoelectric coupling. The device under test, DUT, is placed between two Helmholtz coils that provide the AC magnetic field. The larger coil can provide a DC bias. A lock-in amplifier in differential mode extracts the voltage developed over the capacitor plates upon applying the AC magnetic field.

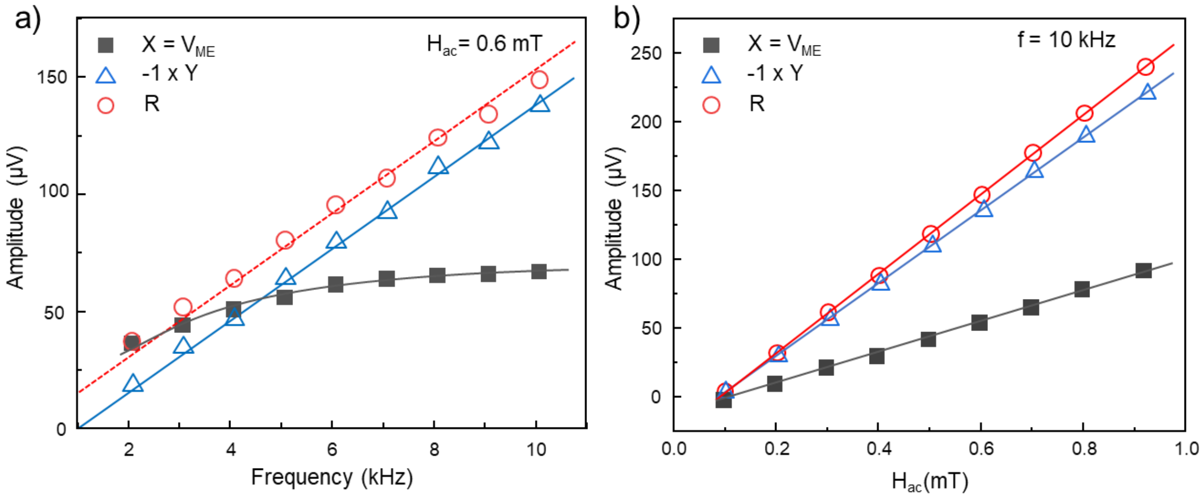

Figure S16. Different components of the lock-in amplifier signals. (a) Frequency dependence of the amplitude of the lock-in amplifier signal and its real and imaginary components. (b) Dependence of the amplitude of the lock-in amplifier signal and its real and imaginary components to the amplitude of the applied magnetic field, H_{ac}.

Lock-in amplifiers produce an amplitude R and phase θ of the signal in the form:

$$Z = Re^{i\theta} \quad \text{where} \quad R = \sqrt{X^2 + Y^2}, \text{ and } \theta = \arctan\left(\frac{Y}{X}\right)$$

with the absolute value $|Z| = R$ given as the amplitude of the signals and θ given by the phase of the input signal relative to the reference signal. The value for θ close to ±90° indicates the dominance of inductive contribution, whereas a value approaching zero indicates the dominance of the magnetoelectric contribution. Any values for θ between these two limits indicate that the signal is a mixture of both magnetoelectric and inductive responses. Therefore, the correct value that should be assigned to the magnetoelectric response of the device is $Rcos\theta$, which is the X component of the signal measured by the lock-in amplifier.

The imaginary part of the signal is due to the inductive contribution and has linear dependencies with both ω. Note that the dependencies of V_{ME} to ω cannot be determined *a priori* and is determined experimentally. The V_{ME} should exhibit frequency dependence because it originates from the response of the piezoelectric phase, whose piezoelectric coefficient and dielectric constant nonlinearly depend on the excitation frequency.

In reporting the magnetoelectric coupling, we consistently reported the X component (or $Rcos\theta$), which accurately measures V_{ME}. Nevertheless, both real and imaginary components of the demodulated signal were recorded. In Figure S12, we have plotted a typical example of the measurement for X, Y, and R for a fixed $H_{DC} = 0.5$ T and H_{ac}values of 0.6 mT. The imaginary component, Y, is negative and has a perfectly linear relationship with the applied frequency, as expected from magnetic induction. The magnetoelectric voltage, X-component of the signal, shows starkly different behavior with a much weaker frequency dependence that tends to saturate at high frequencies, in sharp contrast to the Y component that linearly increases. Reporting R instead of X leads to a substantially over-estimated coupling coefficient due to the contribution of the magnetic induction.

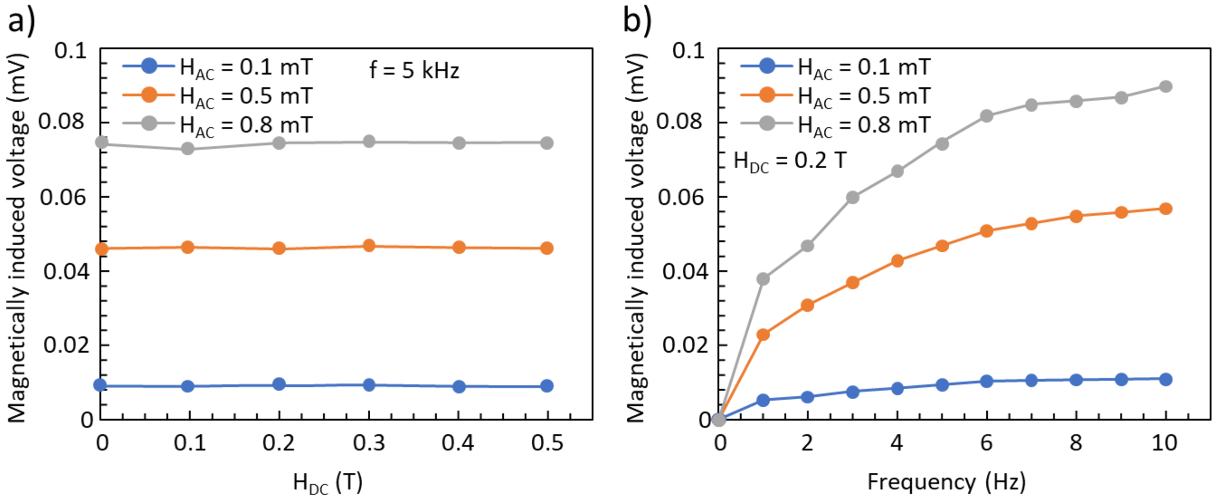

Figure S17. Frequency, H_{AC}, and H_{DC} dependence of α_{ME}. (a) Magnetically induced voltages for a thin-film device with a thickness of 1 µm at different frequencies and amplitude of H_{AC}, at a fixed H_{DC}=0.2 T. (b) Magnetically induced voltage at different H_{DC}s for different H_{AC}s at a fixed frequency of 5 kHz. The symbols show that within the experimental uncertainties, similar coupling coefficients independent of an external DC magnetic field have been obtained. Values are obtained for the nanocomposite with 20 wt% PMMA-grafted nanoparticles.

Table S1.

Overview of the magnetoelectronic (ME) coefficient of different polymer-based multiferroic composites.

Composite	α_{ME} (mV Oe^{-1} cm^{-1})	M_r (emu g^{-1})	P_r (μC cm^{-2})	Ref
P(VDF-TrFE)/$CoFe_2O_4$(NPs)	40	28	6.8	6
P(VDF-TrFE)/$CoFe_2O_4$ (NPs)	41.3	7	7.5	7
P(VDF-TrFE)/$No_{0.5}Zn_{0.5}Fe_2O_4$(NPs)	1.35	N/R*	8	8
Polyurethane/Fe_3O_4(NPs)	11.4	N/R	N/R	9
Polyurethane/Ni (NPs)	6	N/R	8	8
PVDF/$CoFe_2O_4$ (NPs)	11.2	7.8	N/R	10
P(VDF-TrFE)/$CoFe_2O_4$ (NPs)	0.04	7	N/R	11
Dimines 2CN&OCN/$CoFe_2O_4$(NPs)	0.8	4.5	N/R	12
P(VDF-TrFE)/$CoFe_2O_4$ (NPs)	6.5	N/R	N/R	13
P(VDF-TrFE)/Fe_3O_4 (NPs)	0.8	4.5	N/R	12
$BaTiO_3$/$Zn_{0.2}Mn_{0.8}Fe_2O_4$	0.16	0.01	0.25	14
Polyurethane/Fe_3C (NWs)	2.4	N/R	N/R	15
P(VDF-TrFE)/Ni(NWs)	2.3	N/R	2.4	16
P(VDF-TrFE)/FeO(OH) (nanosheet)	0.4	N/R	N/R	13
P(VDF-TrFE)/$SmFeO_3$	45	N/R	2.4	17
PVDF/$(Bi_{0.5}Ba_{0.25}Sr_{0.25})(Fe_{0.5}Ti_{0.5})O_3$	18.4	<0.01	0.5	18
PVDF/$MnFe_2O_4$	6.4	0.009	0.25-0.35	19
PVDF/$BiFeO_3$-$CoFe_2O_4$	22.18	6	5	20
PVDF/$BaTiO_3$	22.2	N/R	9.38	21
PVDF/CNT/$CoFe_2O_4$	16.7	16	N/R	22
P(VDF-TrFE)/ Terfenol-D	38	5	N/R	23
P(VDF-TrFE)/$CoFe_2O_4$	18.5	29	N/R	24
P(VDF-TrFE)/ $Fe_{72.5}Si_{12.5}B_{15}$	65	N/R	N/R	25
P(VDF-TrFE)/ $Zn_{0.2}Co_{0.8}Fe_2O_4$	17	N/R	N/R	26
P(VDF-TrFE)/ $NiFe_2O_4$	90	1.7	5.8	27
P(VDF-TrFE)/CoFe2O_4	35	N/R	4.1	28
P(VDF-TrFE)/$CoFe_2O_4$	47	5.2	5	29
P(VDF-TrFE)/$CoFe_2O_4$	15	6	N/R	30
P(VDF-TrFE)/$CoFe_2O_4$	35	N/R	N/R	31
P(VDF-TrFE)/$Zn_{0.25}Co_{0.75}Fe_2O_4$	16	38	N/R	24

* N/R: Not Reported

References:

1. Galeotti, F.; Bertini, F.; Scavia, G.; Bolognesi, A. A controlled approach to iron oxide nanoparticles functionalization for magnetic polymer brushes. *J. Colloid Interface Sci.* **2011**, 360, 540-547.

2. Fischer, H. The persistent radical effect: a principle for selective radical reactions and living radical polymerizations. *Chem. Rev.* **2011**, 101, 3581-3610.

3. Patten, T. E.; Matyjaszewski, K. Atom transfer radical polymerization and the synthesis of polymeric materials. *Adv. Mater.***1998**, 10, 901-915.

4. Ohno, K.; Akashi, T.; Huang, Y.; Tsujii, Y. Surface-initiated living radical polymerization from narrowly size-distributed silica nanoparticles of diameters less than 100 nm. *Macromolecules* **2010**, 43, 8805-8812.

5. Katsouras, I.; Asadi, K.; Li, M.; van Driel, T. B.; Kjær, K. S.; Zhao, D.; Lenz, T.; Gu, Y.; Blom, P. W. M.; Damjanovic, D.; Nielsen, M. M.; de Leeuw, D. M. The negative piezoelectric effect of the ferroelectric polymer poly(vinylidene fluoride). *Nat. Mater.* **2016**, 15**,** 78-84.

6. Zhang, J. X,; Dai, J. Y.; So, L. C.; Sun, C. L.; Lo, C. Y.; Or, S. W.; Chan, H. L. W. The effect of magnetic nanoparticles on the morphology, ferroelectric, and magnetoelectric behaviors of CFO/P (VDF-TrFE) 0–3 nanocomposites. *J. Appl. Phys.* **2009**, 105, 054102-054106.

7. Martins, P.; Lasheras, A.; Gutiérrez, J.; Barandiaran, J. M.; Orue, I.; Lanceros-Mendez, S. Optimizing piezoelectric and magnetoelectric responses on $CoFe_2O_4$/P (VDF-TrFE) nanocomposites. *J. Phys. D: Appl. Phys.* **2011**, 44**,** 495303-495307.

8. Martins, P.; Moya, X.; Phillips, L.; Kar-Narayan, S.; Mathur, N.; Lanceros-Mendez, S. Linear anhysteretic direct magnetoelectric effect in $Ni_{0.5}Zn_{0.5}Fe_2O_4$/poly (vinylidene fluoride-trifluoroethylene) 0-3 nanocomposites. *J. Phys. D: Appl. Phys.* **2011**, 44**,** 482001-482004.

9. Guyomar, D.; Guiffard, B.; Belouadah, R.; Petit, L. Two-phase magnetoelectric nanopowder/polyurethane composites. *J. Appl. Phys.* **2008**, 104, 074902-074907.

10. Martins, P.; Moya, X.; Caparrós, C.; Fernandez, J.; Mathur, N.; Lanceros-Mendez, S. Large linear anhysteretic magnetoelectric voltage coefficients in $CoFe_2O_4$/polyvinylidene fluoride 0–3 nanocomposites. *J. Nanopart. Res.* **2013**, 15, 1825-1830.

11. Martins, P.; Gonçalves, R.; Lanceros-Mendez, S.; Lasheras, A.; Gutiérrez, J.; Barandiarán, J. Effect of filler dispersion and dispersion method on the piezoelectric and magnetoelectric response of $CoFe_2O_4$/P(VDF-TrFE) nanocomposites. *Appl. Surf. Sci.* **2014**, 313, 215-219.

12. Maceiras, A.; Martins, P.; Gonçalves, R.; Botelho, G.; Ramana, E. V.; Mendiratta, S.; San Sebastián, M.; Vilas, J. L.; Lanceros-Mendez, S.; León, L. M. High-temperature polymer based magnetoelectric nanocomposites. *Eur. Polym. J.* **2015**, 64, 224-228.

13. Martins, P.; Larrea, A.; Gonçalves, R.; Botelho, G.; Ramana, R.; Mendiratta, S.; Sebastian, V.; Lanceros-Mendez, S. Novel anisotropic magnetoelectric effect on δ-FeO (OH)/P (VDF-TrFE) multiferroic composites. *ACS Appl. Mater. Interfaces* **2015**, 7, 11224-11229.

14. Lee, M. K.; Nath, T. K.; Eom, C. B. ; Smoak, M. C. ; Tsui, F. Strain modification of epitaxial perovskite oxide thin films using structural transitions of ferroelectric $BaTiO_3$ substrate. *Appl. Phys. Lett.* **2000**, 77, 3547-3549.

15. Fiorido, T.; Galineau, J.; Salles, V.; Seveyrat, L.; Belhora, F.; Cottinet, P. J. ; Hu, L.; Liu, Y.; Guiffard, B.; Bogner-Van De Moortele, A.; Epicier, T.; Guyomar, D.; Brioude, A. Bifunctional organic/inorganic nanocomposites for energy harvesting, actuation and magnetic sensing applications. *Sen. and Actuators A: Phys.* **2014**, 211, 105-114.

16. Nguyen, T. H. L.; Laffont, L.; Capsal, J. F.; Cottinet, P. J.; Lonjon, A.; Dantras, E.; Lacabanne, C. Magnetoelectric properties of nickel nanowires-P (VDF–TrFE) composites. *Mater. Chem. Phys.* **2015**, 153, 195-201.

17. Ahlawat, A.; Satapathy, S.; Shirolkar, M. M.; Li, J.; Khan, A. A.; Deshmukh, P.; Wang, H. ; Choudhary, R. J. ; Karnal, A. K. Tunable magnetoelectric nonvolatile memory devices based on $SmFeO_3$/P(VDF-TrFE) nanocomposite films. *ACS Appl. Nano. Mater.* **2018**, 1, 3196-3203.

18. Behera, C.; Choudhary, R.; Das, P. Development of multiferroic polymer nanocomposite from PVDF and $(Bi_{0.5}Ba_{0.25}Sr_{0.25})(Fe_{0.5}Ti_{0.5})O_3$ *J. Mater. Sci. Mater. Electron.* **2017**, 28, 2586-2597.

19. Behera, C.; Choudhary, R. Electrical and multiferroic characteristics of PVDF-MnFe2O4 nanocomposites. *J. Alloys Compd.* **2017**, 727, 851-862.

20. Adhlakha, N.; Yadav, K. ; Truccato, M. ; Rajak, P. ; Battiato, A. ; Vittone, E. Multiferroic and magnetoelectric properties of BiFeO3-CoFe2O4-poly (vinylidene-flouride) composite films. *Eur. Polym. J.* **2017**, 91, 100-110.

21. Mayeen, A. ; Kala, M. ; Jayalakshmy, M. ; Thomas, S. ; Rouxel, D. ; Philip, J. ; Bhowmik, R. N. ; Kalarikkal, N. Dopamine functionalization of $BaTiO_3$: an effective strategy for the enhancement of electrical, magnetoelectric and thermal properties of $BaTiO_3$-PVDF-TrFE nanocomposites. *Dalton Trans.* **2018**, 47, 2039-2051.

22. Jing, W. ; Fang, F. A flexible multiferroic composite with high self-biased magnetoelectric coupling. *Compos. Sci. Technol.* **2017**, 153, 145-150.

23. Brito-Pereira, R. ; Ribeiro, C. ; Lanceros-Mendez, S. ; Martins, P. Magnetoelectric response on Terfenol-D/P (VDF-TrFE) two-phase composites. *Compos. B Eng.* **2017**, 120, 97-102.

24. Omelyanchik, A.; Antipova, V.; Gritsenko, C. ; Kolesnikova, V. ; Murzin, D. ; Han, Y. ; Turutin, A. D. ; Kubasov, I. V. ; Kislyuk, A. M. ; Ilina, T. S. ; Kiselev, D. A. ; Voronova, M. I. ; Malinkovich, M. D. ; Parkhomenko, Y. N. ; Silibin, M. ; Kozlova, E. N. ; Peddis, D. ; Levada, K. ; Makarova, L. ; Amirov, A. ; Rodionova, V. Boosting Magnetoelectric Effect in Polymer-Based Nanocomposites *Nanomaterials* **2021**, 11**,** 1154.

25. Polícia, R.; Lima, A. C. ; Pereira, N. ; Calle, E. ; Vázquez, M.; Lanceros-Mendez, S. ; Martins, P. Transparent Magnetoelectric Materials for Advanced Invisible Electronic Applications. *Adv. Electron. Mater.* **2019**, 5, 1900280.

26. Chakraborty, S. ; Mandal, S. K. ; Saha, B. Magneto-optic and magnetoelectric effects in poly(vinylidene fluoride) - $Zn_0Co_{0.8}Fe_2O_4$ nanocomposite organic flexible film *Ceram. Int.* **2019**, 45, 14851–14858.

27. Mayeen, A. ; Kale, M. S. ; Jayalakshmy, M. S.; Thomas, S.; Philip, J.; Rouxel, D.; Bhowmik, R. N. ; Kalarikkal, N. Flexible and self-standing nickel ferrite–PVDF-TrFE cast films: promising candidates for high-end magnetoelectric applications. *Dalton Trans.* **2019**, 48, 16961-16973.

28. Feng, R.; Zhu, Z.; Liu, Y.; Song, Sh.; Zhang, Y.; Yuan, Y.; Han, T.; Xiong, Ch.; Dong, L. Magnetoelectric effect in flexible nanocomposite films based on size-matching, *Nanoscale* **2021**, 13, 4177-4187.

29. Mu, X.; Zhang, H.; Zhang, Ch.; Yang, Sh.; Huang, Y.; Xu, J.; Zhang, Y.; Wang, Q. Li, X. ; Cao, D.; Li, Sh. Poly(vinylidene fluoride-trifluoroethylene)/cobalt ferrite composite films with a self-biased magnetoelectric effect for flexible AC magnetic sensors. *J. Mater. Sci.* ***2021***, 56**,** 9728–9740.

30. Martins, P.; Nunes, J. S.; Oliveira, J.; Peřinka, N.; Lanceros-Mendez, S. Spray-printed magnetoelectric multifunctional composites. *Compos. B: Eng.* **2020**, 187, 107829.

31. Lima, A. C.; Pereira, N.; Ribeiro, C.; Lanceros-Mendez, S.; Martins, P. Greener Solvent-Based Processing of Magnetoelectric Nanocomposites. *ACS Sustainable Chem. Eng.* **2022**, **10**, 4122-4132.

32. Ohno, K.; Morinaga, T.; Takeno, S.; Tsujii, Y.; Fukuda, T., Suspensions of silica particles grafted with concentrated polymer brush: a new family of colloidal crystals. *Macromolecules* **2006,** *39*, 1245-1249.

33. Choi, J.; Hui, C. M.; Pietrasik, J.; Dong, H.; Matyjaszewski, K.; Bockstaller, M. R., Toughening fragile matter: mechanical properties of particle solids assembled from polymer-grafted hybrid particles synthesized by ATRP. *Soft Matter* **2012,** *8*, 4072-4082.

34. Ohno, K.; Morinaga, T.; Koh, K.; Tsujii, Y.; Fukuda, T., Synthesis of monodisperse silica particles coated with well-defined, high-density polymer brushes by surface-initiated atom transfer radical polymerization. *Macromolecules* **2005,** *38*, 2137-2142.

35. Ohno, K.; Akashi, T.; Huang, Y.; Tsujii, Y., Surface-initiated living radical polymerization from narrowly size-distributed silica nanoparticles of diameters less than 100 nm. *Macromolecules* **2010,** *43*, 8805-8812.

36. Pietrasik, J.; Hui, C. M.; Chaladaj, W.; Dong, H.; Choi, J.; Jurczak, J.; Bockstaller, M. R.; Matyjaszewski, K., Silica-Polymethacrylate Hybrid Particles Synthesized Using High-Pressure Atom Transfer Radical Polymerization. *Macromolecular rapid communications* **2011,** *32*, 295-301.

37. Ohno, K.; Morinaga, T.; Takeno, S.; Tsujii, Y.; Fukuda, T., Suspensions of silica particles grafted with concentrated polymer brush: Effects of graft chain length on brush layer thickness and colloidal crystallization. *Macromolecules* **2007,** *40*, 9143-9150.
